# Indoxylsulfate, a Metabolite of the Microbiome, Has Cytostatic Effects in Breast Cancer via Activation of AHR and PXR Receptors and Induction of Oxidative Stress

**DOI:** 10.3390/cancers12102915

**Published:** 2020-10-10

**Authors:** Zsanett Sári, Edit Mikó, Tünde Kovács, Anita Boratkó, Gyula Ujlaki, Laura Jankó, Borbála Kiss, Karen Uray, Péter Bai

**Affiliations:** 1 Department of Medical Chemistry, Faculty of Medicine, University of Debrecen, Egyetem tér 1., 4032 Debrecen, Hungary; sari.zsanett@med.unideb.hu (Z.S.); miko.edit@med.unideb.hu (E.M.); kovacs.tunde@med.unideb.hu (T.K.); boratko@med.unideb.hu (A.B.); ujlaki.gyula@med.unideb.hu (G.U.); janko.laura@med.unideb.hu (L.J.); karen.uray@med.unideb.hu (K.U.); 2 MTA-DE Lendület Laboratory of Cellular Metabolism, 4032 Debrecen, Hungary; 3 Department of Oncology, Faculty of Medicine, University of Debrecen, 4032 Debrecen, Hungary; bkiss@medunideb.hu; 4 Research Center for Molecular Medicine, Faculty of Medicine, University of Debrecen, 4032 Debrecen, Hungary

**Keywords:** breast cancer, microbiome, oncobiome, oncobiosis, indoxylsulfate, EMT, oncometabolism, cancer stem cell, oxidative stress, nitrosative stress, metastasis

## Abstract

**Simple Summary:**

The aim of our study was to identify metabolites of bacterial origin that play role in the pathogenesis of breast cancer. We identified indoxylsulfate, a metabolite of the amino acid tryptophan, as a metabolite with cytostatic properties on breast cancer cells. Cytostatic properties were dependent on increasing oxidative stress blocking the capacity of cancer cells to migrate, enter blood vessels and to form metastases. Furthermore, indoxylsulfate reduced the proportions of cancer stem cells that are highly resistant to chemotherapy and have vital role in initiating recurrence. We identified that indoxylsulfate exert its effects through the pregnane-X receptor and the aryl-hydrocarbon receptor. The expression of these receptors decrease with the progression of the disease, furthermore, the expression of these receptors is low in cases with poor prognosis.

**Abstract:**

Changes to bacterial metabolite-elicited signaling, in oncobiosis associated with breast cancer, plays a role in facilitating the progression of the disease. We show that indoxyl-sulfate (IS), a tryptophan metabolite, has cytostatic properties in models of breast cancer. IS supplementation, in concentrations corresponding to the human serum reference range, suppressed tumor infiltration to the surrounding tissues and metastasis formation in a murine model of breast cancer. In cellular models, IS suppressed NRF2 and induced iNOS, leading to induction of oxidative and nitrosative stress, and, consequently, reduction of cell proliferation; enhanced oxidative and nitrosative stress are crucial in the subsequent cytostasis. IS also suppressed epithelial-to-mesenchymal transition vital for suppressing cellular movement and diapedesis. Furthermore, IS rendered cells hypometabolic, leading to a reduction in aldehyde-dehydrogenase positive cells. Pharmacological inhibition of the pregnane-X receptor using CH223191 and the aryl-hydrocarbon receptor using ketoconazole diminished the IS-elicited effects, suggesting that these receptors were the major receptors of IS in these models. Finally, we showed that increased expression of the human enzymes that form IS (Cyp2E1, Sult1A1, and Sult1A2) is associated with better survival in breast cancer, an effect that is lost in triple negative cases. Taken together, IS, similar to indolepropionic acid (another tryptophan metabolite), has cytostatic properties and higher expression of the metabolic machinery responsible for the formation of IS supports survival in breast cancer.

## 1. Introduction

Oncobiosis refers to the transformation of the microbiome in patients with neoplastic diseases. Several studies demonstrated oncobiosis in the distal gut [1,2,3,4,5,6,7,8,9,10,11,12,13,14,15,16,17,18], the breast microbiome [19,20,21,22,23,24], and oral and urinary microbiomes [22] in breast cancer patients. A majority of the reports show decreased diversity in breast cancer patients compared to controls [1,2,3,4,5,8,9,12,13,14,15,18]. Antibiotic use can suppress diversity, and in murine experimental models of breast cancer, the combination of Vancomycin, Neomycin, Metronidazole, Amphotericin, and Ampicillin aggravated the disease [25,26]. This observation is supported by suggestive supportive evidence in human population studies [27,28,29,30,31,32,33,34]. Furthermore, probiotic treatment may be protective against the incidence of breast cancer [35,36,37,38]. These observations suggest that oncobiosis has a role in the pathomechanism of breast cancer.

The molecular mechanism through which oncobiosis contributes to oncogenesis in breast cancer is poorly characterized. Currently available data suggest that oncobiosis supports carcinogenesis in breast cancer but has little or no role in initiating oncogenesis. Breast cancer oncobiosis modulates the immune system, including lymphocytes and mast cells [16,25,32,39,40,41,42,43]. Furthermore, oncobiosis supports epithelial-to-mesenchymal transition (EMT) [16,17,40,44], migration and invasion [17,40], the reduction of oxidative stress [40,45], the proportions of ALDH1+ cancer stem cells [17,40], and widespread metabolic alterations [16,17,40] in cancers cells. These elementary steps are translated into enhanced tumor growth [16,17], aggressive tumor infiltration to the surrounding tissues [16,17], and enhanced metastasis formation [16,17,41,46,47].

In addition to their immunomodulatory properties, bacteria can secrete metabolites that are absorbed from the gut and carried to tumors through the circulation [48,49]. Among these metabolites, short-chain fatty acids, lithocholic acid, cadaverine, and indole propionic acid, have cytostatic properties [16,17,40,48,50]. These metabolites have pleiotropic effects and can block multiple features of the carcinogenic processes. In breast cancer, the metabolic capacity of the microbiome is suppressed [4,16,17,40,51], suggesting that the production of cytostatic metabolites decrease, probably resulting in reduced bioavailability of bacterial metabolites in the serum and, consequently, in the tumor [52].

Indoxyl-sulfate (IS) is a metabolite of tryptophan [53]. Bacterial metabolism converts tryptophan to indole [53,54,55], which subsequently enters the systemic circulation. Indole is hydroxylated by Cyp2e1 and sulfated by SULT1 and SULT2 enzymes in the liver [53]. The resulting IS reenters the circulation and is excreted through the kidneys. Indole-derivatives can activate the aryl hydrocarbon receptor [53,56] and the pregnane X receptor [40,57]. Tryptophan catabolism affects breast cancer and high extracellular tryptophan levels are associated with worse survival in breast cancer (Table S8 [58]). An indole metabolite, indole propionic acid, has cytostatic properties in breast cancer [40]. In addition, the indole derivative, IS is downregulated both in estrogen receptor-positive and -negative cases ([59]; Additional File 3; Table S3, line 44). Furthermore, in breast tumors, there is a negative correlation between Ki67 positivity (a proliferation marker) and IS levels ([59]; Additional File 9; Table S8, line 130). These data suggest that the involvement of IS in breast cancer is likely. Therefore, we assessed the molecular determinants of IS in the development of breast cancer.

## 2. Results

### 2.1. Indoxyl Sulfate Reduces the Severity of Breast Cancer In Vivo

**Figure 1:**
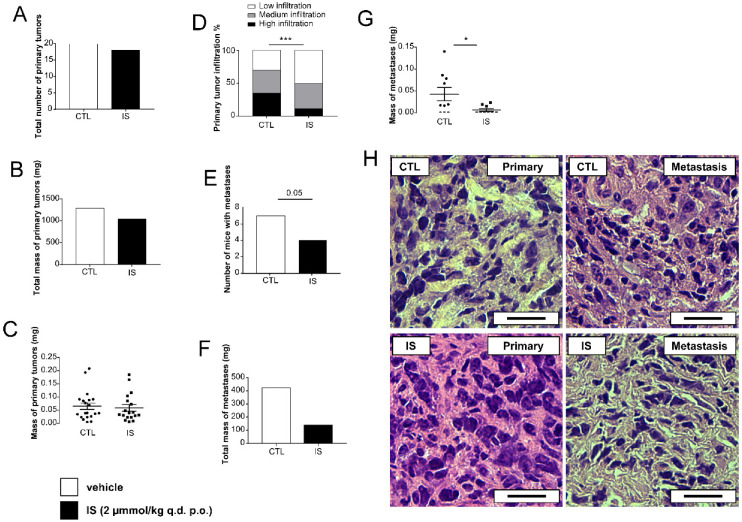
Indoxyl-sulfate (IS) treatment in tumor-bearing mice reduces the infiltration capacity of tumors to the surrounding tissues and reduces metastatic capacity. Female Balb/c mice were grafted with 4T1 cells and treated with IS (2 μmol/kg q.d. p.o.) or vehicle (VEH) (*n* = 10/10) for 14 days before sacrifice. Upon autopsy, (**A**) the total number of primary tumors (20 mice were in each group) and the (**B**) total and (**C**) individual mass of primary tumors were measured. (**D**) The tumor infiltration rates into the surrounding tissues were scored. (**E**) The number of mice with metastases (10 mice were in each group), (**F**) the total mass of metastases, and (**G**) the individual mass of metastases are shown. (**H**) Typical hematoxilin-eosine stained histology sections are displayed. Scale bar equals 40 μm. Numerical values are represented as mean ± SEM. Statistical significance was calculated using Student’s *t*-test (two-tailed) except for panel **D**, where a Chi-square test was used. * and *** indicate statistically significant differences between vehicle and IS groups at *p* < 0.05 and *p* < 0.001, respectively. The whole western blot images of Figure 1 please find in Supplementary Materials Figure S1.

Balb/c female mice were grafted with 4T1 breast cancer cells. Half of the mice received vehicle (sterilized tap water) as a control, while the other half received IS (2 μmol/kg bodyweight). The IS dose corresponds to a 4 μM serum concentration similar to the serum reference range of IS in humans [60]. Per os IS treatment did not inhibit tumor growth (Figure 1A–C), however, IS significantly reduced the infiltration of the primary tumor to the surrounding tissues (Figure 1D). Furthermore, IS treatment reduced the number and mass of metastases (Figure 1E–G). Histology of the primary and the metastatic tumors were not different (Figure 1H).

### 2.2. Indoxyl Sulfate Treatment Inhibits the Proliferation of Breast Cancer Cells

**Figure 2:**
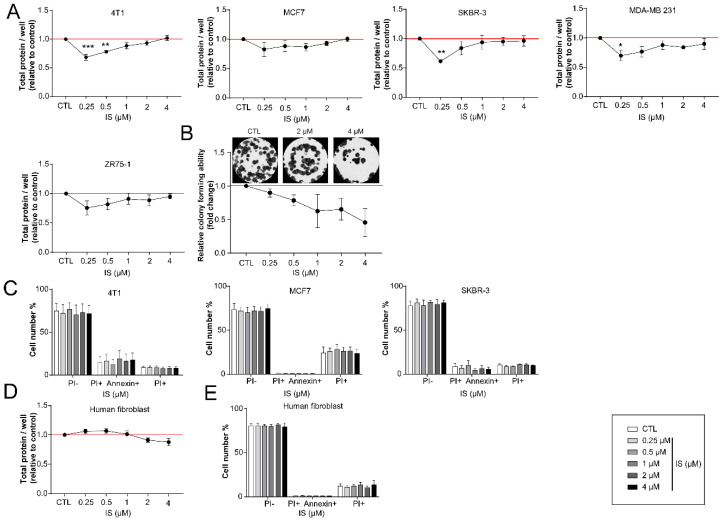
IS has cytostatic properties without affecting cell death. (**A**) 4T1 (1500 cells/well), MCF7 (4000 cells/well), SKBR-3 (5000 cells/well), MDA-MB 231 (3000 cells/well), ZR75-1 (3000 cells/well)were seeded in 96-well plates and treated with IS at the concentrations indicated for 24 h. Total protein content was evaluated using Sulphorhodamine B assays (*n* = 3). (**B**) 4T1 cells (500 cells/well) were seeded in six-well plates and treated with IS at the indicated concentrations for seven days. Colonies were stained according to May-Grünwald-Giemsa and counted using ImageJ software (*n* = 3). (**C**) 4T1 (75,000 cells/well); MCF7 (150,000 cells/well); SKBR-3 (200,000 cells/well), cells were treated with IS in the concentrations indicated for 24 h. The ratios of necrotic and apoptotic cells were determined by double staining with propidium-iodide and FITC Annexin, using the V/Dead Cell Apoptosis Kit, and subjected to flow cytometry (*n* = 3). (**D**) Human fibroblasts (7500 cells/well) were seeded in 96-well plates and treated with IS at the concentrations indicated for 24 h. Total protein content was evaluated using Sulphorhodamine B assays (*n* = 3). (**E**) Human fibroblasts (200,000 cells/well) were seeded in 6-well plates and treated with the indicated concentrations of IS for 24 h. The ratios of necrotic and apoptotic cells were determined by double staining with propidium-iodide and FITC Annexin, using the V/Dead Cell Apoptosis Kit, and subjected to flow cytometry (*n* = 3). Numerical values are represented as mean ± SEM. Fold data were log2 transformed to achieve normal distribution. Statistical significance was determined on panel (**A**,**B**,**D**) by one-way ANOVA followed by Dunnett’s post-hoc tests; all samples were compared to controls. On panels C, and E two-way ANOVAs were conducted followed by Tukey’s post-hoc tests. *, **, and *** indicate statistically significant differences between control and treated samples at *p* < 0.05, *p* < 0.01, and *p* < 0.001, respectively.

IS, similar to other cytostatic metabolites [16,17,40,45,61,62], reduced proliferation in multiple cell lines (SRB assays in 4T1, MCF7, SKBR-3, MDA-MB-231, ZR75-1) at lower concentrations (Figure 2A). The anti-proliferative effects were confirmed in 4T1 cells using clonogenic assays (Figure 2B). The proportions of apoptotic and necrotic cells in culture did not change significantly after treating with IS concentrations corresponding to the human reference concentrations (Figure 2C). IS had no effects on non-transformed, primary human-skin-derived fibroblasts (Figure 2D).

### 2.3. Indoxyl Sulfate Inhibits Numerous Hallmarks of Cancer

**Figure 3:**
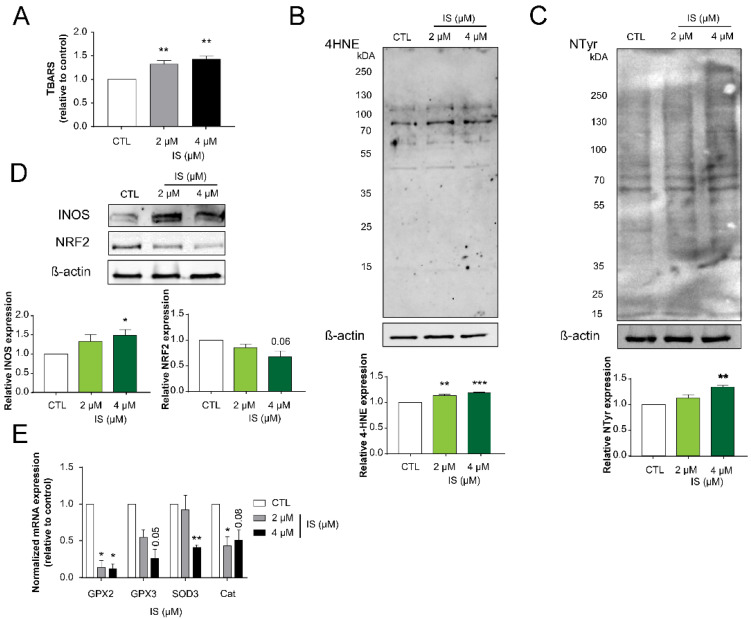
Indoxyl sulfate treatment induces oxidative and nitrosative stress. (**A**) 4T1 cells (500,000 cells/well) were treated with IS at the concentrations indicated for 24 h. Lipid peroxidation was measured using TBARS assays (*n* = 3) and (**B**) 4HNE expression was determined by Western blotting (representative figure, *n* = 3). (**C**) Nitrotyrosine was detected by Western blotting (representative figure, *n* = 3). In the same cells (**D**) the protein levels of iNOS and NRF2 were determined by Western blotting (representative figure, *n* = 3). (**E**) The mRNA expression levels of the indicated genes were determined by RT-qPCR (*n* = 3). Numerical values are represented as mean ± SEM. Fold data were log2 transformed to achieve normal distribution. Statistical significance was determined using ANOVA followed by Dunnett’s post-hoc test, where all values were compared to control. *, **, and *** indicate statistically significant differences between control and treated samples at *p* < 0.05, *p* < 0.01, and *p* < 0.001, respectively. Abbreviations: thiobarbituric acid reactive substances (TBARS); 4-hydroxynoneal (4HNE); nitro-tyrosine (NTyr); inducible nitric oxide synthase (iNOS); nuclear factor 2 (NRF2); glutathione peroxidase 2 (GPX2); glutathione peroxidase 3 (GPX3); superoxide dismutase 3 (SOD3); catalase (CAT). For 4HNE and NTyr blots whole lanes were subject to densitometry, while for iNOS, NRF2 and actin the bands of interest was subject to densitometry. The whole western blot images of Figure 3 please find in Figure S1.

We assessed whether IS can modulate cancer hallmarks that were modulated by other cytostatic bacterial metabolites [16,17,41,47,61,62,63,64,65]. First, we assessed oxidative/nitrosative stress markers, as these are major regulators of cancer hallmarks and cancer progression [66,67,68,69,70]. Levels of thiobarbituric acid-reactive substances (TBARS) and 4-hydroxynonenal (4HNE), both markers of lipid oxidative damage, increased when cells were treated with IS (Figure 3A,B). Next, we assessed nitro-tyrosine (NTyr) levels that indicate protein damage and nitrosative stress [71,72]. NTyr levels were induced by IS treatment (Figure 3C) suggesting increased damage to cells by reactive species and damage to cellular lipids and proteins. These changes correlated with the increased expression of inducible nitric oxide synthase (iNOS) (Figure 3D) and the decreased expression of glutathione peroxidase 2 and 3 (GPX1 and GPX3), superoxide dismutase 3 (SOD3), and catalase (cat) (Figure 3D,E).

**Figure 4:**
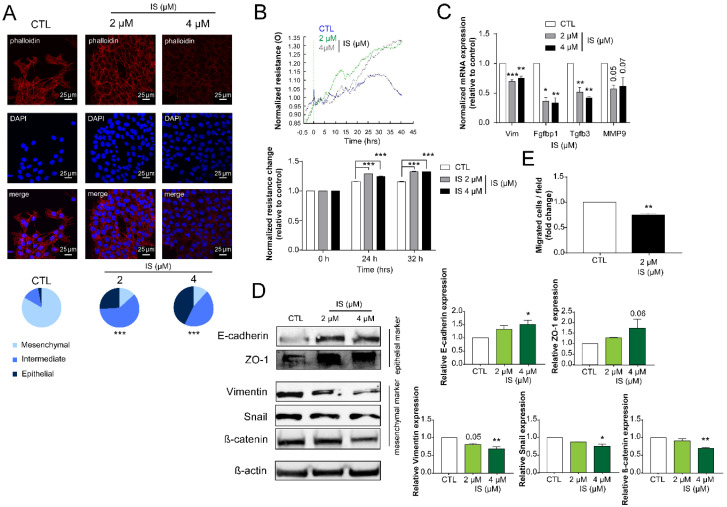
IS treatment induces mesenchymal-to-epithelial transition and blocks cellular migration. (**A**) 4T1 cells (100,000 cells/well) were treated with IS in the concentrations indicated for 24 h then cellular morphology was observed using Texas Red-X Phalloidin and DAPI staining (representative figure, *n* = 3). Scale bar corresponds to 25 μm. (**B**) Total impedance was measured by ECIS (representative figure, mean ± SD, *n* = 1). (**C**,**D**) After IS treatment of 4T1 cells, the expressions of the indicated genes were determined using (**C**) RT-qPCR *(n* = 3) and (**D**) Western blotting (representative figure, *n* = 3). *β*-actin was used as a loading control. (**E**) 4T1 cells (50,000 cells/well) were treated with the indicated concentration of IS for 24 h and, subsequently, the percentages of migrated cells were determined using a Corning Matrigel invasion chamber (*n* = 3). Cells were counted by the Opera Phoenix High Content Screening System using Harmony 4.6 Software (Perkin-Elmer, Waltham MA, USA). Numerical values are represented as mean ± SEM, except for panel B, where mean ± SD was plotted. Statistical significance was determined using ANOVA followed by Dunnett’s post-hoc tests, except for panel A, where a Chi-square test was conducted. For Dunnett’s tests, all comparisons were made to controls. *, **, and *** indicate statistically significant differences between control and treated samples at *p* < 0.05, *p* < 0.01, and *p* < 0.001, respectively.

The 4T1 cells reverted the epithelial-to-mesenchymal transition (EMT) after IS treatment, evidenced by dose-dependent conversion of 4T1 cells to epithelial morphology (Figure 4A), coinciding with increased resistance (Figure 4B). In good agreement with these findings, the mRNA and protein expression of mesenchymal markers (vimentin (Vim), fibroblast growth factor-binding protein 1 (Fgfbp1), transforming growth factor beta-3 (Tgfb3), matrix metalloproteinase 9 (MMP9), snail family transcriptional repressor-1 (SnaiI), and β-catenin decreased, while the expression of epithelial markers (E-cadherin and tight junction protein-1 (ZO-1) increased (Figure 4C,D). In addition, cells migrated less in a Boyden-chamber experiment (Figure 4E).

**Figure 5:**
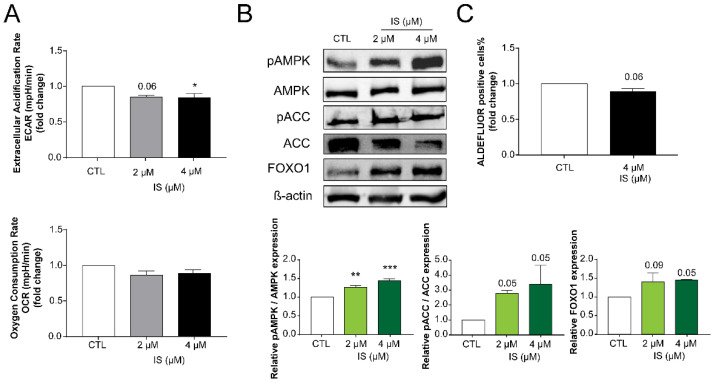
IS treatment renders cells metabolically less flexible and reduces the proportions of ALDH1-positive cells. (**A**) 4T1 cells (2000 cells/well) were treated with IS in the concentrations indicated for 24 h then cells were subjected to a Seahorse XF96 analysis. The mitochondrial oxygen consumption rate (OCR) and extracellular acidification rate (ECAR) were measured and plotted (*n* = 3). (**B**) The expression levels of the indicated proteins were determined by Western blotting (*n* = 3). *β*-actin was used as a loading control. (**C**) 4T1 cells (100,000 cells/well) were treated with the indicated concentration of IS for 24 h then the proportions of aldehyde dehydrogenase-positive cells were measured by Aldefluor assay using flow cytometry (*n* = 3). Numerical values are represented as mean ± SEM. Fold data were log2 transformed to achieve normal distribution. Statistical significance was determined using ANOVA followed by Dunnett’s post-hoc tests, except for C panel, where a Student’s *t*-test (two-tailed) was used. For the Dunnett’s post-hoc tests, all comparisons were made to controls. *, **, and *** indicate statistically significant differences between control and treated samples at *p* < 0.05, *p* < 0.01, and *p* < 0.001, respectively. Abbreviations: phospho-AMP-activated protein kinase (pAMPK); AMP-activated protein kinase (AMPK); phospho-Acetyl Co-A Carboxylase (pACC); Acetyl Co-A Carboxylase (ACC) and Forkhead box protein O1 (FOXO1). The whole western blot images of Figure 5 please find in Figure S1.

IS treatment had a profound effect on cellular metabolism. IS treatment reduced extracellular acidification rate (ECAR) rendering cells hypometabolic and metabolically less flexible (Figure 5A). Surprisingly, the hypometabolic switch coincided with the induction of key energy sensors, including AMPK phosphorylation or FOXO1 expression (Figure 5B). AMPK activation upon IS treatment was marked by phosphorylation of its alpha subunit on Thr172 and the phosphorylation of a key AMPK target protein, ACC on Ser79 (Figure 5B). Finally, the proportions of ALDH1-positive cancer stem cells decreased upon treatment with IS (Figure 5C), a feature that is linked to changes in cellular metabolism [73,74].

### 2.4. IS Exerts Its Effects through the AHR and PXR Receptors

**Figure 6:**
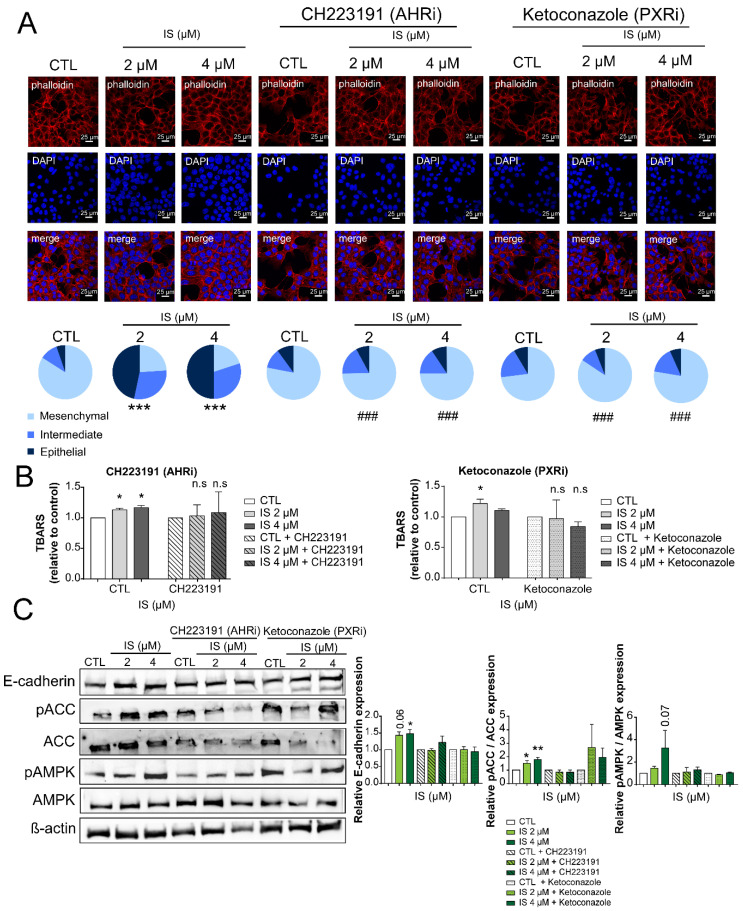
Pharmacological inhibition of aryl hydrocarbon receptor (AHR) and pregnane-X-receptor (PXR) block IS-elicited antineoplastic effects. (**A**) 4T1 cells (100,000 cells/well) were treated with IS in the concentrations indicated for 24 h with or without the inhibitors, as indicated. The actin cytoskeleton and nuclei were stained using Texas Red-X Phalloidin and DAPI, then the morphology was assessed using Leica PP8 confocal system (representative figure, *n* = 3). On the same cells, (**B**) lipid peroxidation (TBARS assays) (*n* = 3) was measured. (**C**) Expression of the indicated proteins was determined by Western blotting (representative figure, *n* = 3). *β*-actin was used as a loading control. Numerical values are represented as mean ± SEM. Fold data were log2 transformed to achieve normal distribution. Statistical significance was determined using ANOVA followed by Dunnett’s post-hoc tests, except for panel C, where a Student’s *t*-test (two-tailed) was used. For Dunnett’s tests, all comparisons were made to controls. *, **, and *** indicate statistically significant differences between control and treated samples at *p* < 0.05, *p* < 0.01, and *p* < 0.01, respectively. Abbreviations: non-significant (ns); PXR inhibitor (PXRi); AHR inhibitor (AHRi); phospho-AMP-activated protein kinase (pAMPK); AMP-activated protein kinase (AMPK); phospho-Acetyl Co-A Carboxylase (pACC); Acetyl Co-A Carboxylase (ACC).

As the next step, we wanted to assess which receptors are responsible for the IS-elicited effects. Indoxyl derivatives exert their effects through the aryl hydrocarbon receptor (AHR) and pregnane-X-receptor (PXR) [53]. We applied pharmacological inhibitors to interrogate the involvement of AHR and PXR in IS signaling. The AHR inhibitor, CH223191, and the PXR inhibitor, ketoconazole, were applied [75,76]. Both CH223191 and ketoconazole blocked IS-elicited mesenchymal-to-epithelial transition (Figure 6A). Inhibition of AHR and PXR also attenuated the IS-induced increases in TBARS (Figure 6B). In addition, IS-induced expression of E-cadherin was blocked by CH223191, but not by ketoconazole (Figure 6C). In contrast, the phosphorylation of ACC and AMPK was blocked by both agents (Figure 6C).

### 2.5. Higher Expression of the Isoforms of SULT and Cyp2e1 Correlate with Better Survival in Breast Cancer

**Figure 7:**
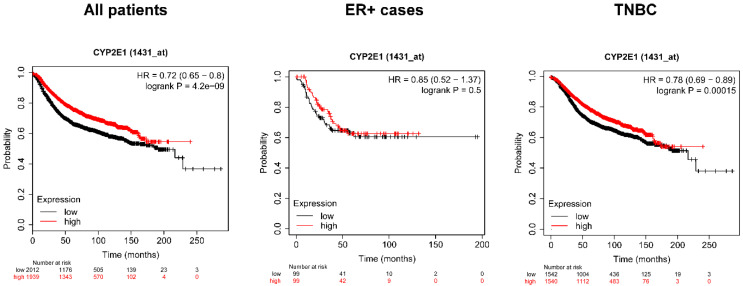
Higher expression of *Cyp2E1* prolongs survival in breast cancer patients. The effect of expression of *Cyp2E1* on survival in breast cancer was analyzed by kmplot.com, a freely accessible database. The data depicted stems from data acquired from microarray experiments and patients were stratified as a function of receptor expression. Total survival rates were assessed, and all samples are represented. The database was assessed on 30 March 2020.

**Figure 8:**
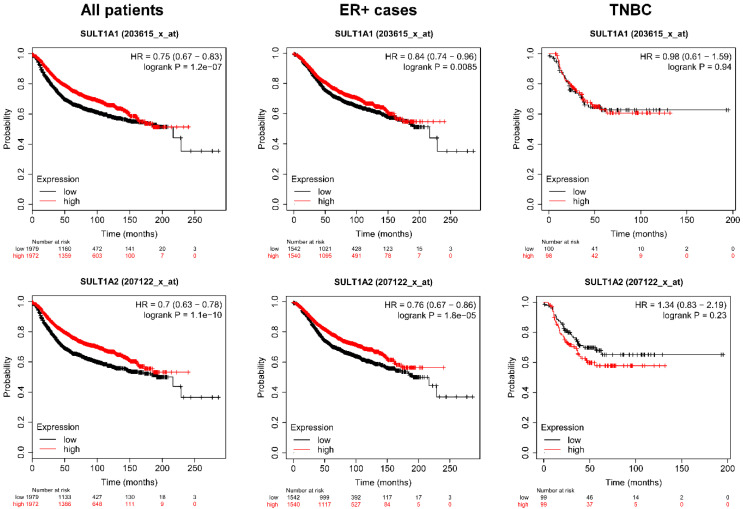
Higher expression of *Sult1A1* or *Sult1A2* prolongs survival in breast cancer patients. The effect of expression of *Sult1A1* and *Sult1A2* on survival in breast cancer was analyzed by kmplot.com, a freely accessible database. The data depicted stems from data acquired from microarray experiments and patients were stratified as a function of receptor expression. Total survival rates were assessed, and all samples are represented. The database was assessed on 30 March 2020.

Previously, we showed that higher expression of AHR and PXR, the receptors for IS prolong survival in breast cancer patients [40]. Subsequently, we assessed how the expression of IS biosynthesis enzymes affect the survival of breast cancer patients. To that end, we assessed an online database, kmplot.com [77]. Higher expression of *Cp2E1* and *Sult1A1* and *Sult1A2* in tumors correlated with better survival in breast cancer patients (Figure 7 and Figure 8, Table 1, Table 2 and Table 3). Furthermore, similar to other bacterial cytostatic metabolites [16,17,40,45], the protective effect was lost in triple negative cases (TNBC) (Figure 7 and Figure 8, Table 1, Table 2 and Table 3).

The effects of Cyp2E1 expression on survival in breast cancer were analyzed by kmplot.com, a freely accessible database. Total survival rates were assessed, and all samples are represented in different subpopulations of breast cancer. Numbers in bold represent statistically significant results. The database was accessed on the 30 March 2020.

The effects of Sult isoform expression on survival in breast cancer were analyzed by kmplot.com, a freely accessible database. Total survival rates were assessed, and all samples are represented in different subpopulations of breast cancer. Numbers in bold represent statistically significant results. The database was accessed on the 30 March 2020.

## 3. Discussion

In this study, we identified a novel bacterial metabolite, IS, that possesses cytostatic features in breast cancer, similar to the short-chain fatty acids [61,62], lithocholic acid [16,45,50,78,79,80,81], cadaverine [17,51], and indole propionic acid [40]. Despite the conflicting results, the majority of breast cancer microbiome studies show suppressed diversity and biosynthetic capacity in breast cancer-associated oncobiosis [1,2,3,4,5,8,9,12,13,14,15,48], leading to limited biosynthetic capacity that lowers the level of protective bacterial metabolites in patients [16,52]. Lower biosynthetic capacity is associated with ER cases [40,51] or triple negative cases [9,12,13] that have worse clinical outcomes. In addition, the application of antibiotics, which suppress microbial diversity, increases the risk for breast cancer in human and animal studies [25,26,27,28,29,30,31,32,33,34,82]. On the other hand, probiotic treatment, which enriches the gut microbiome, decreases the incidence of breast cancer [35,36,37,38]. These results demonstrate that oncobiotic transformation in breast cancer has pathological relevance that is further supported by nutritional studies [83]. We have previously shown that the indole-derivative biosynthetic capacity is suppressed the most in the early stages of breast cancer (in situ carcinoma and stage 1) [40].

Previously, we showed that tryptophan catabolism is suppressed in breast cancer [40]. Suppressed tryptophan catabolism is associated with worse survival in human breast cancer (Table S8) [58]. Furthermore, IS was shown to be downregulated in breast cancer patients ([59]; Additional File 3; Table S3, line 44) and there is a negative correlation between the Ki67 positivity (proliferation) and 3-indoxyl sulfate levels ([59]; Additional File 9; Table S8, line 130). Taken together, the results presented here can be translated to human breast cancer, whereby, IS production is suppressed in breast cancer patients and is associated with poor outcomes.

IS exert its effects through the AHR and PXR receptors [53], and AHR seems to be the more dominant receptor. Indolepropionic acid, another tryptophan metabolite with cytostatic effects in breast cancer, seems to have more balanced effects on both receptors [40]. Lower AHR and PXR expression correlate with higher disease stage, grade, and enhanced mitotic activity in the tumor [40]. Apparently, indole-induced signaling is gradually lost as breast cancer evolves.

IS administration reduces cell proliferation, infiltration into the surrounding tissues, and metastasis formation in cellular and animal models. This feature is a common trait for other cytostatic metabolites in breast cancer [16,17,40,45,50,51,61,62,78,79,80,81]. At the same time, IS did not affect non-transformed cells, again a common trait for bacterial cytostatic metabolites in breast cancer [16,17,40,78], suggesting that these metabolites have tumor cell-specific effects.

The underlying molecular mechanism involved in IS effects on tumor progression is the reversal of the epithelial-to-mesenchymal transition, similar to other metabolites [16,17,40,41,44,45,47]. Reverting or inhibiting EMT slows cell movement, diapedesis, and metastasis formation, as was observed in our study. In conjunction with the decreased EMT, we observed decreased expression of mesenchymal markers (Vim, Fgfbp1, Tgfb3, MMP9, SnaiI, β-catenin) and a concomitant upregulation in epithelial markers (E-cadherin and ZO-1). Suppressed EMT may be the leading cause of the suppressed cellular movement, diapedesis, and metastasis formation.

We also observed increased oxidative and nitrosative stress due to increased iNOS expression and suppressed NRF2 activation. These changes are key elements for cytostasis in breast cancer, and occur with the indole derivative, indole propionic acid, also [45,69,84,85,86]. Increases in reactive species suppress the proportions of cancer stem-cells [73,87,88,89] and contribute to cytostasis [40,45]. Very likely, the metabolic alterations also contribute to cytostasis and to suppressing cancer stem cells [73,74,90].

From a broader perspective, our findings fit the puzzle of the pathomechanism of human breast cancer, as tryptophan and indole metabolism are tightly related to breast cancer and breast cancer survival [40,91,92]. Previously we showed that bacterial tryptophan metabolism is suppressed in early stages of breast cancer, releasing the brake on breast cancer cells [16,17,40]. In that sense, IS behaves similar to other cytostatic metabolites [16,17,25,32,39,40,41,42,43,45,46]. Of note, in addition to the loss of cytostatic properties, the breast cancer oncobiome has an increased capacity for reactive estrogens and increase estrogen enterohepatic circulation [1,93]. Consequently, parent estrogens, estrone and estradiol, boost cell proliferation, and their catechol-quinone metabolites cause oxidative DNA damage and mutagenesis [94]. Taken together, the oncobiome seems to have a role primarily in breast carcinoma progression, but little or no role in the initiation of the disease.

## 4. Materials and Methods

All methods were performed according to the relevant guidelines.

### 4.1. Chemicals

All chemicals, including IS and ketoconazole, were from Sigma-Aldrich (St. Louis, MI, USA) unless otherwise stated. IS was used at concentrations of 2 μM and 4 μM, which correspond to the normal human serum concentration of IS [53,95,96]. The aryl hydrocarbon receptor (AHR) inhibitor, CH223191, was obtained from MedChemExpress (MCE, Monmouth Junction, NJ, USA) and was applied at a concentration of 10 μM. Pregnane X receptor (PXR) downstream signaling was inhibited using ketoconazole at a final concentration of 25 μM [75,76].

### 4.2. Cell Culture

The 4T1 murine breast cancer cells were maintained in RPMI-1640 (Sigma-Aldrich, R5886) medium containing 10% FBS, 1% penicillin/streptomycin, 2 mM L-glutamine, and 1% pyruvate at 37 °C with 5% CO_2_. MCF7 human breast cancer cells were maintained in MEM (Sigma-Aldrich, M8042) medium containing 10% FBS, 1% penicillin/streptomycin, and 2 mM L-glutamine at 37 °C with 5% CO_2_. SKBR-3 human breast cancer cells were maintained in DMEM (Sigma-Aldrich, 1000 mg/L glucose, D5546) medium containing 10% FBS, 1% penicillin/streptomycin, and 2 mM L-glutamine at 37 °C with 5% CO_2_. ZR75-1 human breast cancer cells were maintained in RPMI-1640 (Sigma-Aldrich, R5886) medium containing 10% FBS, 1% penicillin/streptomycin, and 2 mM L-glutamine at 37 °C with 5% CO_2_. Human primary fibroblasts cells were maintained in DMEM (Sigma-Aldrich, 1000 mg/L glucose, D5546) medium containing 20% FBS, 1% penicillin/streptomycin, and 2 mM L-glutamine at 37 °C with 5% CO_2_.

### 4.3. In Vitro Cell Proliferation Assays

Cellular proliferation was assessed using Sulphorhodamine B (SRB) and colony forming assays as described in Miko et al. and Fodor et al. [16,97]. Cells were seeded in 96-well plates (4T1, 1500 cells/well; MDA-MB-231, 3000 cells/well; SKBR-3, 5000 cells/well; MCF7, 4000 cells/well; ZR75-1, 3000 cells/well; human fibroblast, 7500 cells/well) in complete medium and were cultured with different concentrations of IS for 24 h. Then, cells were fixed by the addition of 50% trichloroacetic acid (TCA, final concentration: 10%) and the plates were incubated for 1 h at 4 °C. Plates were washed five times in water and stained with 0.4% (w/v) SRB solution in 1% acetic acid. Unbound dye was removed by washing five times with 1% acetic acid. Bound stain was solubilized with 10 mM Tris base and the absorbance was measured on an automated plate reader (Thermo Labsystems Multiskan MS, Walthman, MA, USA) at 540 nm.

For colony forming assays, cells were seeded in six-well plates (4T1, 500 cells/well) and treated with the indicated concentrations of IS for seven days. After treatment, the plates were washed twice with PBS. Colonies were fixed in methanol for 15 min, dried, and stained with the solution of May-Grünwald-Giemsa for 20 min. Plates were washed with water and the colonies were counted using Image J software [98].

### 4.4. Detection of Cell Death

IS induced cytotoxicity was assessed by simple propidium iodide (PI; Biotium, Fremont, CA, 40016, USA) uptake assays, as described in Kovacs et al. [17]. Cells were seeded in six-well plates (4T1, 75,000 cells/well; MCF7, 150,000 cells/well; SKBR-3, 200,000 cells/well; human fibroblasts, 200,000 cells/well) and treated with the indicated concentrations of IS for 24 h followed by staining with 100 μg/mL PI for 30 min at 37 °C. Adherent cells and supernatants were collected in FACS tubes, washed once with PBS, and analyzed by flow cytometry (FACS Calibur, BD Biosciences).

To evaluate changes in necrotic and apoptotic cell death, we used an Annexin V+PI double staining assay kit (Invitrogen, Carlsbad, CA, USA, V13242). Cells were seeded in six-well plates (4T1, 75,000 cells/well; MCF7, 150,000 cells/well; SKBR-3, 200,000 cells/well; human fibroblast, 200,000 cells/well) and treated with the indicated IS concentrations for 24 h. Then, the collected cells were stained with 100 μg/mL PI solution and 5 μL FITC Annexin V, according to the manufacturer’s instructions. The numbers of apoptotic and necrotic cells were measured using a FACS Calibur flow cytometer.

### 4.5. Electric Cell-Substrate Impedance Sensing (ECIS)

ECIS measurements (ECIS model Zθ, Applied BioPhysics Inc., Troy, NY, USA) were used to monitor cell-to-cell and cell-to-surface connections. The 4T1 cells were seeded (40,000 cells/well) on type 8W10E arrays. Cells were treated with vehicle or 2 μM or 4 μM IS for 20 h and total impedance values were measured for 24 h. Multi-frequency measurements were taken at 62.5, 125, 250, 500, 1000, 2000, 4000, 8000, 16,000, 32,000, and 64,000 Hz. The reference well was set to a no-cell control with complete medium. ECIS assays were performed similar to Miko et al. [16].

### 4.6. Immunocytochemistry

Immunocytochemistry was performed similarly to Miko et al. [16]. The 4T1 cells were grown on glass coverslips for one day and treated with the indicated concentrations of IS and the AHR inhibitor, CH223191 (10 μM), or the PXR inhibitor, ketoconazole (25 μM), for 24 h. Then, cells were washed with PBS, fixed with 4% paraformaldehyde for 15 min, and permeabilized using 1% Triton X-100 in PBS for 5 min. Cells were then blocked with 1% BSA in PBS for 1 h and incubated with TexasRed-X Phalloidin (T7471, 1:150, Invitrogen, Carlsbad, CA, USA) for 1 h at 4 °C. Cell nuclei were visualized with DAPI (R37606, 1:10, Thermo Fischer Scientific Inc., Rockford, IL, USA) and rinsed in PBS twice for 10 min. Coverslips were mounted in Mowiol/Dabco solution. Confocal images were acquired with a Leica TCS SP8 confocal microscope and were processed using LAS AFv3.1.3 software (Wetzlar, Germany). Typical mesenchymal-like and epithelial-like morphology of 4T1 cells are represented in Figure 4A and Figure 6A and in Miko et al. [16]. Epithelial-type cells are more round in shape with the actin cytoskeleton localized below the cell membrane. Mesenchymal-type are elongated, the actin cytoskeleton is organized into fibers aligning with the longer axis of the cell.

### 4.7. mRNA Preparation and Quantitation

Reverse transcription-coupled PCR (RT-qPCR) was performed similar to Szanto et al. [99]. Total RNA from cells was prepared using TRIzol reagent according to the manufacturer’s instructions (Invitrogen Corporation, Carlsbad, CA, USA). For assessing the expression of the indicated genes, 2 μg of RNA was reverse transcribed using High Capacity cDNA Reverse Transcription Kit (Applied Biosystems, Foster City, CA, USA). The qPCR reactions were carried out with the qPCRBIO syGreen Lo-ROX Supermix (PCR Biosystems Ltd., London, UK) on a Light-Cycler 480 Detection System (Roche Applied Science). Gene expression was normalized to the geometric mean of human 36B4 and cyclophyllin values. Primers are listed in Table 4.

### 4.8. Seahorse Metabolic Flux Analysis

Changes in oxygen consumption rate (OCR, reflecting mitochondrial oxidative capacity) and pH, termed extracellular acidification rate (ECAR, reflecting glycolysis) were measured using an XF96 oximeter (Seahorse Biosciences, North Billerica, MA, USA). The 4T1 cells were seeded in 96-well Seahorse assay plates (4T1, 2000 cells/well) and treated with vehicle or the indicated IS concentrations for 24 h. The OCR and ECAR values were recorded every 30 min to monitor the effects of IS treatment. Data were normalized to protein content and normalized readings were used for calculations.

### 4.9. Aldefluor Assay

Aldehyde dehydrogenase (ALDH) activity was determined using an Aldefluor Stem Cell kit (StemCell Technologies, Vancouver, BC, Canada). The 4T1 cells were seeded on six-well plates (4T1, 100,000 cells/well) and treated with the indicated concentrations of IS for 24 h. Then, the collected cells were processed according to the manufacturer’s instructions. The SKBR-3 cell line was used for positive control samples based on the manufacturers’ instructions. Changes in the level of ALDH were assessed by flow cytometry and the results were analyzed using Flowing Software 2.5.1 (Beckton-Dickinson, Franklin Lakes, NJ, USA). The Aldefluor assay for assessing stemness was performed similarly to references [17,100,101].

### 4.10. SDS-PAGE and Western Blotting

Protein isolation, SDS PAGE, and Western blotting were performed as described in Nagy et al. [102]. Cells were lysed in RIPA buffer (50 mM Tris, 150 mM NaCl, 0.1% SDS, 1% TritonX 100, 0.5% sodium deoxycholate, 1mM EDTA, 1mM Na_3_VO_4_, 1 mM PSMF, 1 mM NaF, and protease inhibitor cocktail). Protein extracts (20–50 μg) were separated on 10% SDS polyacrylamide gels and transferred onto nitrocellulose membranes by electroblotting. After blocking for 1 h in TBST containing 5% BSA, the membranes were incubated with primary antibodies overnight at 4 °C. The membranes were washed with 1× TBST solution, then probed with IgG HRP-conjugated peroxidase secondary antibodies (1:2000, Cell Signaling Technology, Inc, Beverly, MA, USA). Bands were visualized by enhanced chemiluminescence (SuperSignal West Pico Solutions, Thermo Fisher Scientific Inc., Rockford, IL, USA). Blots were quantified by densitometry using the Image J software and the results of densitometry is uploaded alongside with the primary data to Figshare.com (https://figshare.com/s/81c2f5906706c60e6c3f). The primary and secondary antibodies are listed in Table 5.

### 4.11. Determination of Lipid Peroxidation

Lipid peroxidation was measured by determining the production rate of thiobarbituric acid-reactive substrate using the thiobarbituric acid-reactive substances (TBARS) assay as described in Mabley et al. [103]. The 4T1 cells were seeded in T75 flasks and exposed to AHR (10 μM) and PXR (25 μM) inhibitors together with IS (2 μM or 4 μM) for 24 h. Cells were rinsed in PBS and scraped, then collected by centrifugation. After adding 8.1% SDS, 20% acetic acid, 0.8% thiobarbituric acid (TBA), and distilled water to the cell pellet, the samples were incubated at 96 °C for 1 h. Samples were cooled on ice and centrifuged. The absorbance of the supernatants was measured at 540 nm. The levels of 4-hydroxynonenal (4HNE)-modified proteins, as a marker for lipid peroxidation, were also assessed using western blotting.

### 4.12. Invasion

Matrigel invasion assays were performed with 4T1 cells using Corning BioCoat Matrigel Invasion Chambers (Corning, NY, USA). Cells were seeded in the chambers (50,000 cells/well) in serum free medium and grown overnight. Then, the cells were exposed to the indicated concentrations of IS for 24 h. The lower chamber was filled with 4T1 medium with 100 ng/mL SDF1-alpha (Sigma-Aldrich, SRP4388) as a chemoattractant. Cells were prepared and stained with Hematoxylin-Eosin (VWR, PA, USA, 340374T 341972Q) dye according to the manufacturer’s instructions. Cells were then analyzed on the Opera Phoenix High Content Screening System using Harmony 4.6 Software. Migration was calculated from the percentage of migrated cells through the Matrigel control membranes.

### 4.13. Animal Study

Animal experiments were authorized by the Institutional Animal Care and Use Committee at the University of Debrecen and the National Board for Animal Experimentation (1/2015/DEMÁB) and were performed according to the NIH guidelines (Guide for the Care and Use of Laboratory Animals) and applicable national laws. Animal studies are reported in compliance with the ARRIVE guidelines.

Experimental animals were BALB/c female mice between 14–16 weeks of age (20–25 g). Mice were randomized for all experiments. Animals were bred in the “specific pathogen-free” zone of the Animal Facility at the University of Debrecen and kept in the “minimal disease” zone during the experiments. Five mice were housed in each cage (standard block shape 365 × 207 × 140 mm, surface 530 cm^2^; 1284 L Eurostandard Type II. L from Techniplast). Cages were changed once a week, on the same day. The dark/light cycle was 12 h and the temperature was 22 ± 1 °C. Mice had ad libitum access to food and water (sterilized tap water). Animals had paper tubes to enrich their environment. The animal facility was overseen by a veterinarian. A total of 20 female mice were used in the study, 10 randomly selected control and 10 IS-fed mice.

The 4T1 cells were suspended (2 × 10^6^/mL) in ice-cold PBS-Matrigel (1:1, Sigma-Aldrich) at a 1:1 ratio. Twenty female BALB/c mice received 50 μL injections to their second inguinal fat pads on both sides (10^5^ cells/injection site). Tumor growth and animal well-being were monitored daily.

IS was administered by oral gavage at a dose of 2 μmol/kg as a bolus once a day. The dose correlated with the serum reference concentration of IS [53,95,96]. IS stock (60 mM) was prepared in sterilize tap water and stored at −20 °C. The IS stock solution was diluted on the day of treatment. Animals were randomized into two groups: 10 mice were treated with IS, and 10 mice were treated with vehicle (sterilized tap water). The researchers administering IS and vehicle solutions were blinded. Treatment was carried out every day at the same time between 09:00 and 11:00. Animals were sacrificed on day 14 post grafting by cervical dislocation, and primary tumors and metastases were harvested for subsequent analysis.

During autopsy, primary tumors were visually assessed and scored based on their infiltration rate into surrounding tissues and the macroscopic appearance of the tumor [16,17]. Tumors were classified as “low infiltration” class if the primary tumor remained in the mammary fat pads without any detectable attachment to muscle. “Medium infiltration” tumors attached to the muscle tissue but did not penetrate the abdominal wall. If the tumor grew into the muscle tissue and penetrated the abdominal wall, the tumor was scored as a “high infiltration” tumor. Researchers involved in scoring primary tumor infiltration rates were blinded. The tumors outside the primary transplantation sites were considered metastases. Both primary and metastatic tumor masses were removed from the animals and measured on an analytical balance in pre-weighed Eppendorf tubes.

### 4.14. Database Screening

The kmplot.com database [77] was used to examine the connection between gene expression levels (Cyp2E1, Sult1A1, and Sult1A2) and breast cancer survival in humans. Probe numbers are listed in Table 3.

### 4.15. Statistical Analysis

For comparing the two groups, we used two-tailed Student’s *t*-tests, unless stated otherwise. Fold data were log_2_ transformed to achieve normal distribution. Statistical significance was determined for multiple comparisons with one-way analysis of variance (ANOVA) followed by Tukey’s or Dunnett’s honest significance difference (HSD) post-hoc test, as stated in the figure captions. All data are presented as mean ± SEM unless otherwise stated. Texas Red-X Phalloidin-labelled fluorescent pictures were analyzed using Cell Profiler 2.0 (github.com/CellProfiler/CellProfiler) followed by Advanced Cell Classifier 3.0 (www.cellclassifier.org). FACS results were analyzed using Flowing Software 2.0. Statistical analysis was done using GraphPad Prism 7 (Graphpad Software Inc., San Diego, CA, USA) software unless stated otherwise.

## 5. Conclusions

In this paper, we showed that indoxylsulfate, an indoxyl-derivative bacterial metabolite that is further metabolized in the liver, has cytostatic properties in breast cancer. In a previous study, we showed that bacterial tryptophan catabolism is suppressed in breast cancer, and in this studym we show that higher expression of the hepatic enzymes producing indoxylsulfate lead to better survival suggesting that the cytostatic properties conferred by indoxylsulfate production are suppressed in breast cancer. Furthermore, the loss of the cytostatic properties are important in the pathophysiology of breast cancer.

## Figures and Tables

**Table 1:** The number of patients at risk for Figure 7 and Figure 8. Values were obtained from the kmplot.com database. The database was accessed on the 30 March 2020.

Patient Group	Probe		Time (Months)						HR
			0	50	100	150	200	250	
**All cancers**	*CYP2E1*	low	2012	1176	505	139	23	3	0.72
	1431_at	high	1939	1343	570	102	4	0	(0.65–0.8)
	*CYP2E1*	low	1980	1177	482	133	16	2	0.68
	209975_at	high	1971	1342	593	108	11	1	(0.61–0.76)
	*CYP2E1*	low	2091	1360	633	160	23	2	0.96
	209976_s_at	high	1860	1159	442	81	4	1	(0.86–1.07)
	*CYP2E1*	low	1984	1171	487	113	12	0	0.78
	222100_at	high	1967	1348	588	128	15	3	(0.7–0.87)
	*SULT1A1*	low	1979	1160	472	141	20	3	0.75
	203615_x_at	high	1972	1359	603	100	7	0	(0.67–0.83)
	*SULT1A1*	low	1976	1150	463	139	22	3	0.77
	215299_x_at	high	1975	1369	612	102	5	0	(0.69–0.86)
	*SULT1A2*	low	1979	1133	427	130	18	3	0.7
	207122_x_at	high	1972	1386	648	111	9	0	(0.63–0.78)
	*SULT1A2*	low	1976	1149	440	133	18	3	0.73
	211385_x_at	high	1975	1370	635	108	9	0	(0.66–0.82)
**ER+ cases**	*CYP2E1*	low	1542	1004	436	125	19	3	0.78
	1431_at	high	1540	1112	483	76	3	0	(0.69–0.89)
	*CYP2E1*	low	1542	1019	430	119	15	2	0.74
	209975_at	high	1540	1097	489	82	7	1	(0.65–0.85)
	*CYP2E1*	low	1622	1125	531	130	19	2	0.94
	209976_s_at	high	1460	991	388	71	3	1	(0.83–1.07
	*CYP2E1*	low	1606	1052	451	106	13	1	0.82
	222100_at	high	1476	1064	468	95	9	2	(0.72–0.93)
	*SULT1A1*	low	1542	1021	428	123	15	3	0.84
	203615_x_at	high	1540	1095	491	78	7	0	(0.74–0.96)
	*SULT1A1*	low	1542	1009	421	123	17	3	0.87
	215299_x_at	high	1540	1107	498	78	5	0	(0.76–0.98)
	*SULT1A2*	low	1542	999	392	117	17	3	0.76
	207122_x_at	high	1540	1117	527	84	5	0	(0.67–0.86)
	*SULT1A2*	low	1541	1004	390	111	16	3	0.81
	211385_x_at	high	1541	1112	529	90	6	0	(0.71–0.92)
**Triple negative cases**	*CYP2E1*	low	99	41	10	2	0		0.85
	1431_at	high	99	42	9	0	0		(0.52–1.37)
	*CYP2E1*	low	100	46	6	2	0		0.96
	209975_at	high	98	37	13	0	0		(0.59–1.55)
	*CYP2E1*	low	99	33	5	0	0		0.8
	209976_s_at	high	99	50	14	2	0		(0.49–1.3)
	*CYP2E1*	low	99	42	12	2	0		1.17
	222100_at	high	99	41	7	0	0		(0.72–1.9)
	*SULT1A1*	low	100	41	10	2	0		0.98
	203615_x_at	high	98	42	9	0	0		(0.61–1.59)
	*SULT1A1*	low	99	40	5	2	0		1.18
	215299_x_at	high	99	43	14	0	0		(0.72–1.91)
	*SULT1A2*	low	99	46	14	2	0		1.34
	207122_x_at	high	99	37	5	0	0		(0.83–2.19)
	*SULT1A2*	low	99	42	12	2	0		1.06
	211385_x_at	high	99	41	7	0	0		(0.66–1.72)

**Table 2:** Link between Cyp2E1 expression and breast cancer patient survival.

Patient Group	*CYP2E1* (1431_at)		*CYP2E1* (209975_at)		*CYP2E1* (209976_s_at)		*CYP2E1* (222100_at)	
	HR	*p*-Value	HR	*p*-Value	HR	*p*-Value	HR	*p*-Value
All breast Cancers *n* = 3951	0.72	***	0.68	***	0.96	0.460	0.78	***
ER(+), PR(+), *n* = 577	1.29	0.170	1.02	0.920	0.76	0.140	0.84	0.340
ER(−), PR(−), *n* = 298	1.06	0.770	1.23	0.300	1.05	0.820	1.04	0.830
ER(−), PR(−), HER2(−) *n* = 198	0.80	0.500	0.96	0.860	0.80	0.370	1.17	0.520
ER(+), Luminal A, *n* = 1933	0.76	**	0.68	***	0.99	0.920	0.81	*
ER(+), Luminal A, Grade 1, *n* = 267	0.97	0.920	1.38	0.300	0.81	0.520	1.27	0.450
ER(+), Luminal B, *n* = 1149	0.79	*	0.81	0.030	0.87	0.160	0.74	**
ER(+), Luminal B, Grade1 *n* = 56	1.46	0.530	1.04	0.950	0.83	0.770	1.24	0.720
Grade1, *n* = 345	1.02	0.940	1.15	0.590	0.76	0.310	1.22	0.450
Grade2, *n* = 901	1.13	0.330	1.13	0.310	0.94	0.640	0.74	*
Grade3, *n* = 903	0.93	0.54	0.94	0.56	1.11	0.330	0.85	0.16
Basal subtype, *n* = 618	0.59	***	0.62	***	0.89	0.360	0.91	0.470
Luminal A, *n* = 1933	0.76	**	0.68	***	0.99	0.920	0.81	*
Luminal B, *n* = 1149	0.79	*	0.81	*	0.87	0.160	0.74	**
ER(+), HER2(+), *n* = 156	1.25	0.470	1.69	0.093	1.14	0.670	1.26	0.460
ER(−), HER2(+), *n* = 96	1.37	0.320	1.58	0.150	1.78	0.068	0.81	0.510
ER(+), PR(+),Lymph(+) *n* = 344	1.41	0.120	1.05	0.810	0.80	0.310	1.02	0.930
ER(+), PR(+),Lymph(−) *n* = 228	0.81	0.560	1.06	0.870	0.76	0.400	0.44	*
ER(−), PR(−),Lymph(+) *n* = 127	1.26	0.390	1.08	0.780	1.16	0.580	1.21	0.470
ER(−), PR(−),Lymph(−) *n* = 167	1.00	1.000	1.11	0.740	0.96	0.900	1.32	0.370
ER(+), Luminal A, Grade 2, *n* = 567	0.91	0.580	1.13	0.460	1.01	0.970	0.89	0.480
ER(+), Luminal B, Grade2 *n* = 253	0.65	0.051	0.82	0.360	0.46	**	0.59	*
ER(+) *n* = 3082	0.78	***	0.74	***	0.94	0.3382	0.82	**

HR—hazard ratio. The *p* values were calculated using a Log Rank test. *, **, and *** indicate statistically significant differences between the lowest and the highest quartile at *p* < 0.05, *p* < 0.01, and *p* < 0.01, respectively.

**Table 3:** Link between SultA1 expression, SultA2 expression, and breast cancer patient survival.

Gene	*SULT1A1* (203615_x_at)		*SULT1A1* (215299_x_at)		*SULT1A2* (207122_x_at)		*SULT1A2* (211385_x_at)	
Patient Group	HR (Hazard Ratio)	*p*-Value (Log Rank Test)	HR (Hazard Ratio)	*p*-Value (Log Rank Test)	HR (Hazard Ratio)	*p*-Value (Log Rank Test)	HR (Hazard Ratio)	*p*-Value (Log Rank Test)
All breast Cancers *n* = 3951	0.72	***	0.77	***	0.70	***	0.73	***
ER(+), PR(+), *n* = 577	1.16	0.430	1.19	0.330	1.18	0.370	1.22	0.270
ER(−), PR(−), *n* = 298	1.37	0.120	1.42	0.085	1.34	0.140	1.24	0.280
ER(−), PR(−), HER2(−) *n* = 198	0.98	0.940	1.18	0.510	1.34	0.230	1.06	0.810
ER(+), Luminal A, *n* = 1933	0.86	0.092	0.93	0.399	0.78	**	0.70	**
ER(+), Luminal A, Grade 1, *n* = 267	0.83	0.560	1.59	0.150	1.01	0.960	1.13	0.700
ER(+), Luminal B, *n* = 1149	0.84	0.075	0.83	0.064	0.68	***	0.84	0.078
ER(+), Luminal B, Grade1 *n* = 56	0.91	0.870	0.89	0.850	1.31	0.660	1.43	0.550
Grade1, *n* = 345	0.94	0.810	1.32	0.300	1.17	0.560	1.14	0.630
Grade2, *n* = 901	0.96	0.730	1.09	0.500	0.84	0.170	0.91	0.430
Grade3, *n* = 903	1.02	0.860	1.00	1.000	0.94	0.58	1.04	0.74
Basal subtype, *n* = 618	0.72	*	0.85	0.210	0.73	*	0.75	*
Luminal A, *n* = 1933	0.86	0.092	0.93	0.400	0.78	**	0.77	**
Luminal B, *n* = 1149	0.84	0.075	0.83	0.064	0.68	***	0.84	0.078
ER(+), HER2(+), *n* = 156	1.24	0.490	1.13	0.690	0.96	0.900	1.18	0.600
ER(−), HER2(+), *n* = 96	1.47	0.230	1.40	0.300	1.31	0.390	1.05	0.880
ER(+), PR(+),Lymph(+) *n* = 344	1.29	0.240	1.23	0.340	1.26	0.290	1.21	0.380
ER(+), PR(+),Lymph(−) *n*=228	0.89	0.720	0.87	0.680	1.08	0.820	1.33	0.380
ER(−), PR(−),Lymph(+) *n* = 127	1.54	0.110	1.22	0.470	1.32	0.310	1.25	0.400
ER(−), PR(−),Lymph(−) *n* = 167	1.41	0.270	1.10	0.760	1.39	0.290	1.12	0.720
ER(+), Luminal A, Grade 2, *n* = 567	1.12	0.500	1.51	*	0.92	0.620	1.06	0.720
ER(+), Luminal B, Grade2 *n* = 253	0.68	0.076	0.63	*	0.78	0.240	0.76	0.200
ER(+) *n* = 3082	0.84	**	0.87	*	0.76	***	0.81	***

HR—hazard ratio. The *p* values were calculated using a Log Rank test. *, **, and *** indicate statistically significant differences between the lowest and the highest quartile at *p* < 0.05, *p* < 0.01, and *p* < 0.01, respectively.

**Table 4:** Murine and human primers used in reverse transcription-coupled PCR (RT-qPCR) reactions.

**Gene Symbol**	**Murine Forward Primer (5′-3′)**	**Murine Reverse Primer (5′-3′)**
CAT	CCTTCAAGTTGGTTAATGCAGA	CAAGTTTTTGATGCCCTGGT
VIM	CTCCAGAGAGAGGAAGCCGAAAG	CCTGGATCTCTTCATCGTGCAGT
FgfBp1	CAAGGTCCAAGAAGCTGTCTCCA	AGCTCCAAGATTCCCCACAGAAC
Tgfb3	GGCGTCTCAAGAAGCAAAAGGAT	CCTTAGGTTCGTGGACCCATTTC
MMP9	CATTCGCGTGGATAAGGAGT	ACCTGGTTCACCTCATGGTC
GPX2	GTTCTCGGCTTCCCTTGC	TTCAGGATCTCCTCGTTCTGA
GPX3	GGCTTCCCTTCCAACCAA	CCCACCTGGTCGAACATACT
SOD3	CTCTTGGGAGAGCCTGACA	GCCAGTAGCAAGCCGTAGAA
Cyclophilin A	TGGAGAGCACCAAGACAGACA	TGCCGGAGTCGACAATGAT
36B4	AGATTCGGGATATGCTGTTGG	AAAGCCTGGAAGAAGGAGGTC
**Gene Symbol**	**Human Forward Primer (5′-3′)**	**Human Reverse Primer (5′-3′)**
AHR	TTGAACCATCCCCATACCCCAC	GAGGTTCTGGCTGGCACTGATA
PXR	AGTGAAGGTTCCCGAGGACATG	TTGTCACAGAGCATACCCAGCA
Cyclophilin A	GTCTCCTTTGAGCTGTTTGCAGAC	CTTGCCACCAGTGCCATTATG
36B4	CCATTGAAATCCTGAGTGATGTG	GTCGAACACCTGCTGGATGAC

**Table 5:** List of antibodies used for Western blotting.

Antibody	Dilution	Vendor
4-HNE	1:1000	Abcam (ab46545)
Nitrotyrosine	1:1000	Millipore (06-284)
iNOS	1:1000	Novus (NB300-605)
NRF2	1:1000	Abcam (ab31163)
Phospho-AMPKα (Thr172)	1:1000	Cell Signaling (#2535)
AMPKα	1:1000	Cell Signaling (#5832)
Phospho-ACC (Ser79)	1:1000	Cell Signaling (#3661)
ACC	1:1000	Cell Signaling (#3676)
FOXO1	1:1000	Cell Signaling (#9454)
E-cadherin	1:1000	Cell Signaling (#3195)
ZO1	1:1000	Cell Signaling (#8193)
Vimentin	1:1000	Cell Signaling (#5741)
Snail	1:1000	Cell Signaling (#3879)
β-Catenin	1:1000	Sigma-Aldrich (C7082)
β-Actin	1:20000	Sigma-Aldrich (A3854)
Anti-rabbit IgG, HRP-linked antibody	1:2000	Cell Signaling (#7074)
Anti-Mouse IgG, Peroxidase antibody	1:2000	Sigma-Aldrich (A9044)

## Data Availability

All primary data is accessible at https://figshare.com/s/81c2f5906706c60e6c3f. The DOI, 10.6084/m9.figshare.12444743 will become active upon acceptance of the manuscript.

## Supplementary Materials

The following are available online at https://www.mdpi.com/2072-6694/12/10/2915/s1, Figure S1: The whole western blot images of Figures 1B, 3C,D, and 5B.
