# Supplementary material for: Indoxylsulfate, a Metabolite of the Microbiome, Has Cytostatic Effects in Breast Cancer via Activation of AHR and PXR Receptors and Induction of Oxidative Stress
Source: Cancers (Basel). 2020 Oct 10;12(10):2915. doi: 10.3390/cancers12102915 (PMC7599465; doi:10.3390/cancers12102915)
Supplement: Supplementary file 1 [file cancers-12-02915-s001.pdf]

cancers

MDPI

Article

Indoxylsulfate, a Metabolite of the Microbiome, has Cytostatic Effects in Breast Cancer via Activation of AHR and PXR Receptors and Induction of Oxidative Stress

Zsanett Sári [1], Edit Mikó [1,2], Tünde Kovács [1], Anita Boratkó [1], Gyula Ujlaki [1], Laura Jankó [1], Borbála Kiss [3], Karen Uray [1], and Péter Bai [1,2,4,*]

1 Department of Medical Chemistry, Faculty of Medicine, University of Debrecen, Egyetem tér 1., 4032 Debrecen, Hungary; sari.zsanett@med.unideb.hu (Z.S.); miko.edit@med.unideb.hu (E.M.); kovacs.tunde@med.unideb.hu (T.K.); boratko@med.unideb.hu (A.B.); ujlaki.gyula@med.unideb.hu (G.U.); janko.laura@med.unideb.hu (L.J.); karen.uray@med.unideb.hu (K.U.)

2 MTA-DE Lendület Laboratory of Cellular Metabolism, 4032 Debrecen, Hungary

3 Department of Oncology, Faculty of Medicine, University of Debrecen, 4032 Debrecen, Hungary; bkiss@medunideb.hu

4 Research Center for Molecular Medicine, Faculty of Medicine, University of Debrecen, 4032 Debrecen, Hungary

* Correspondence: baip@med.unideb.hu, Tel. +36-52-412-345; Fax. +36-52-412-566

Received: 23 September 2020; Accepted: 08 October 2020; Published: date

Supplementary materials

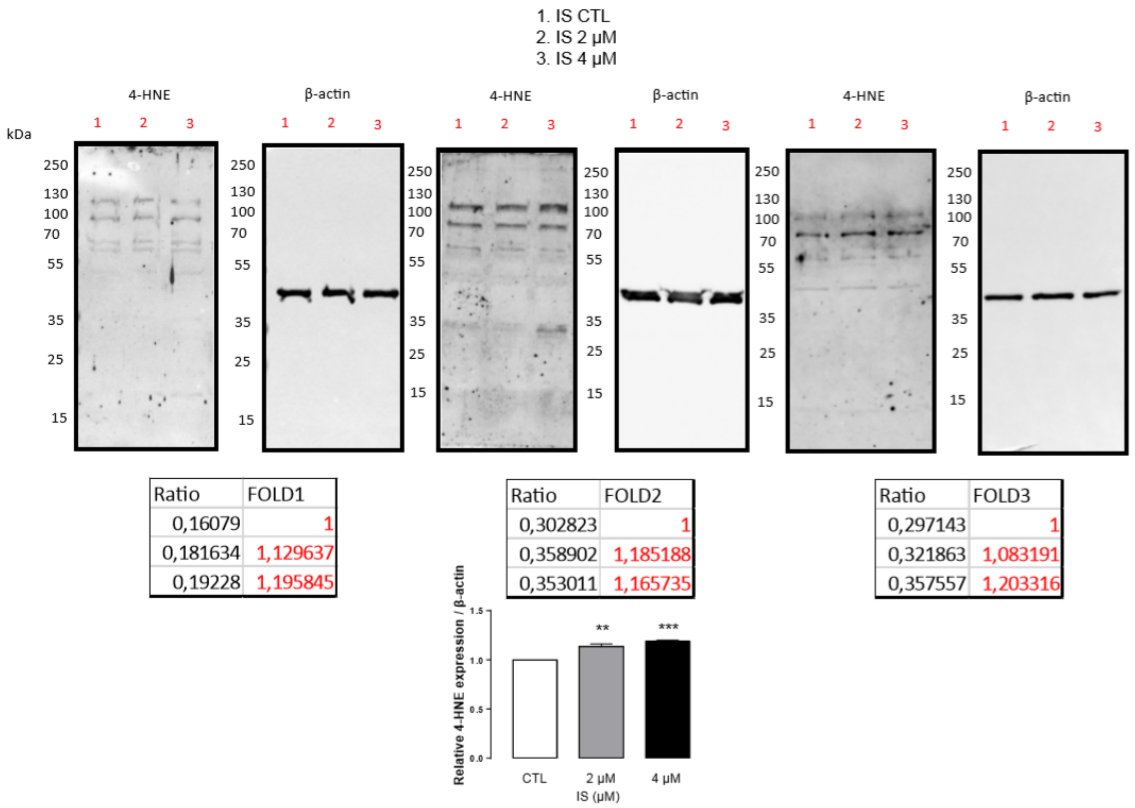

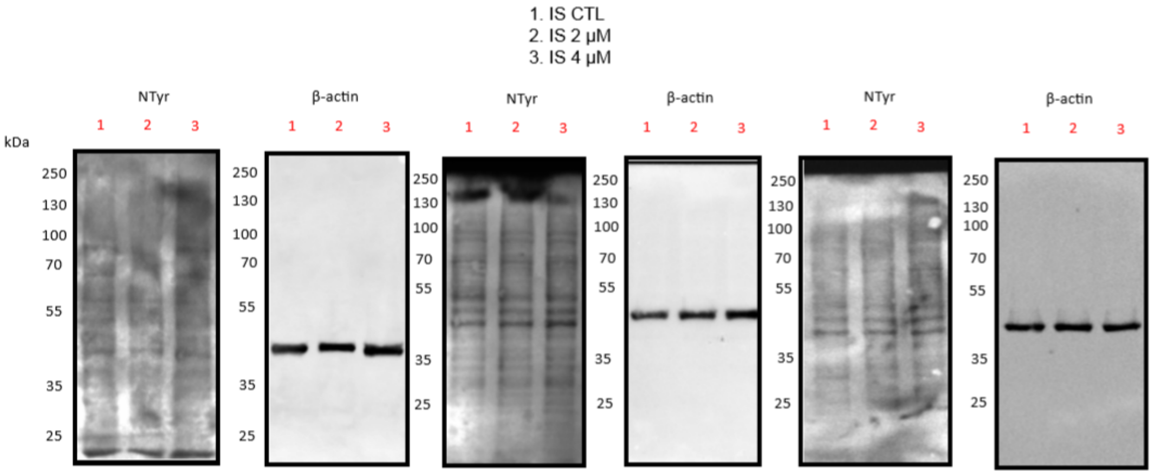

Ratio	FOLD1
0,509564	1
0,513904	1,008515
0,652834	1,281161

Ratio	FOLD2
0,549696	1
0,678364	1,234073
0,747634	1,360088

Ratio	FOLD3
0,373116	1
0,425878	1,14141
0,516645	1,384678

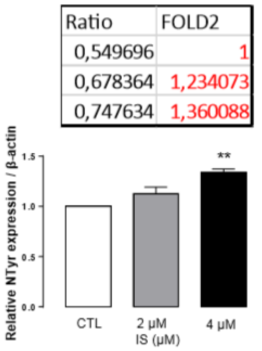

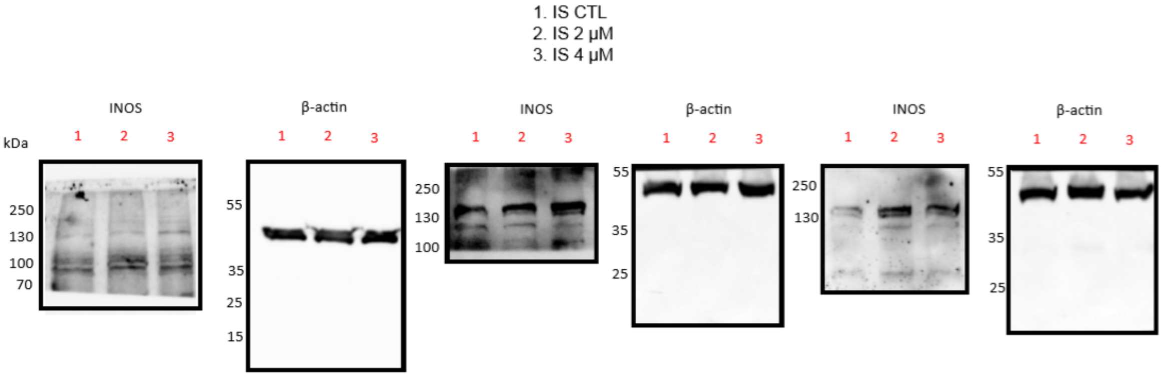

Ratio	FOLD1
0,275405	1
0,309711	1,124564
0,373111	1,354771

Ratio	FOLD2
0,864038	1
1,028351	1,190169
1,124269	1,30118

Ratio	FOLD3
0,462288	1
0,781125	1,689694
0,828917	1,793074

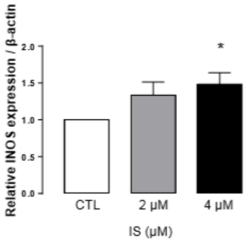

1. IS CTL
2. IS 2 µM
3. IS 4 µM

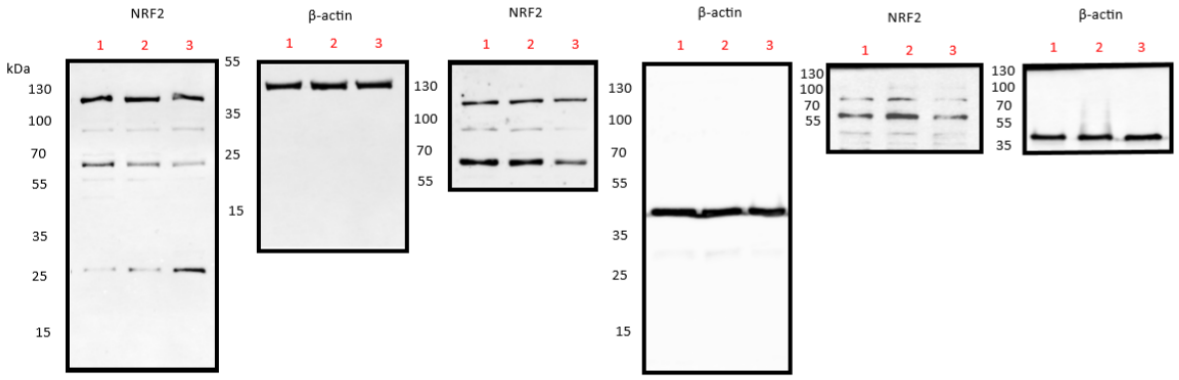

Ratio	FOLD1
0,464795	1
0,336038	0,722983
0,220149	0,473649

Ratio	FOLD2
1,139782	1
1,033949	0,907146
0,97562	0,85597

Ratio	FOLD3
0,719999	1
0,677233	0,940604
0,509257	0,707302

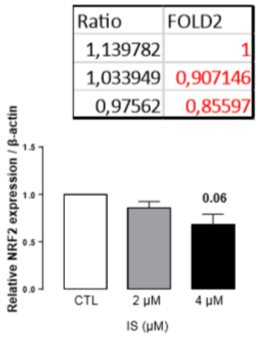

1. IS CTL
2. IS 2 µM
3. IS 4 µM

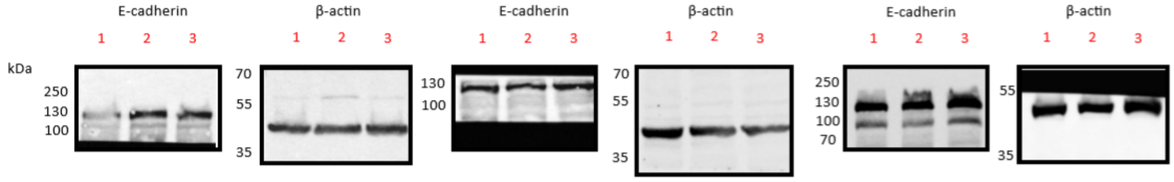

Ratio	
0,683403	1
1,109822	1,623965
1,123173	1,643501

Ratio	FOLD2
0,784392	1
0,899313	1,146509
1,339002	1,707056

Ratio	FOLD3
0,85734	1
1,02784	1,19886
1,03325	1,205179

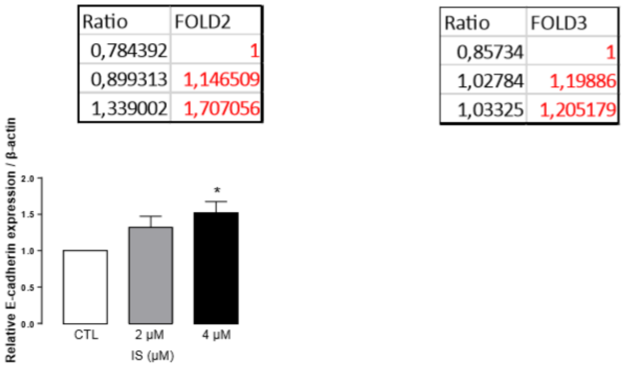

1. IS CTL
2. IS 2 µM
3. IS 4 µM

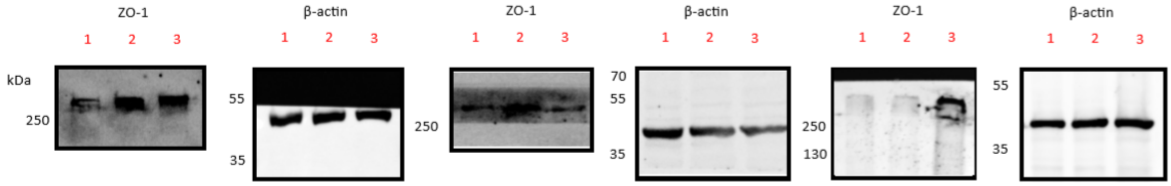

Ratio	FOLD1
0,910652	1
1,208769	1,327367
1,188925	1,305575

Ratio	FOLD2
1,293235	1
1,671337	1,292369
1,708943	1,321448

Ratio	FOLD3
0,261244	1
0,32319	1,237118
0,674834	2,583152

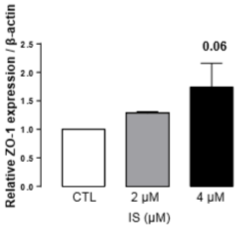

1. IS CTL
2. IS 2 µM
3. IS 4 µM

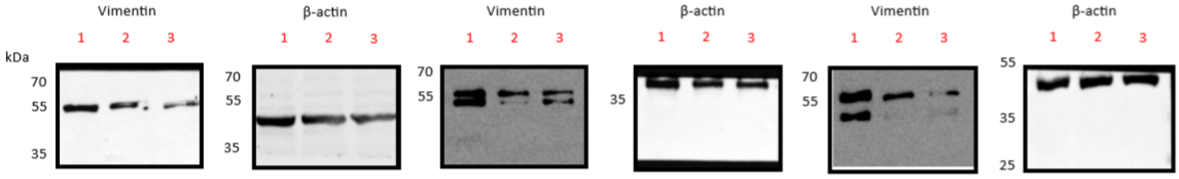

Ratio	FOLD1
1,243959	1
0,923215	0,742159
0,814894	0,655082

Ratio	FOLD2
0,988705	1
0,867638	0,87755
0,781107	0,790031

Ratio	FOLD3
1,489562	1
1,159983	0,778741
0,874179	0,58687

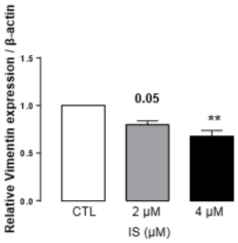

1. IS CTL
2. IS 2 μM
3. IS 4 μM

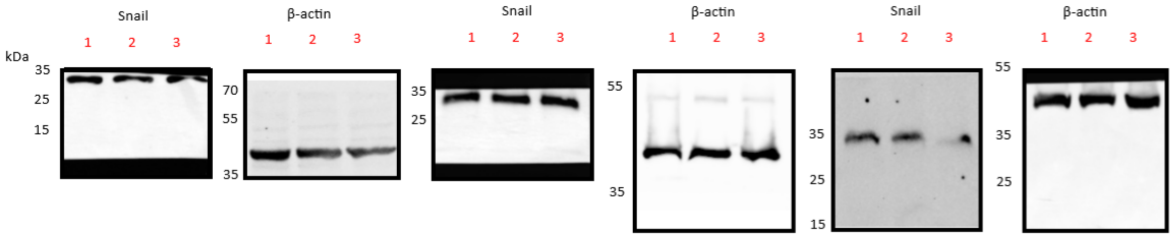

Ratio	FOLD1
1,126607	1
0,97929	0,869238
0,962631	0,854451

Ratio	FOLD2
1,847502	1
1,625449	0,879809
1,420203	0,768715

Ratio	FOLD3
0,700101	1
0,616647	0,880797
0,437507	0,62492

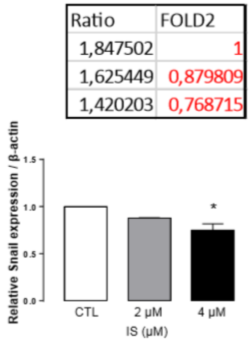

1. IS CTL
2. IS 2 μM
3. IS 4 μM

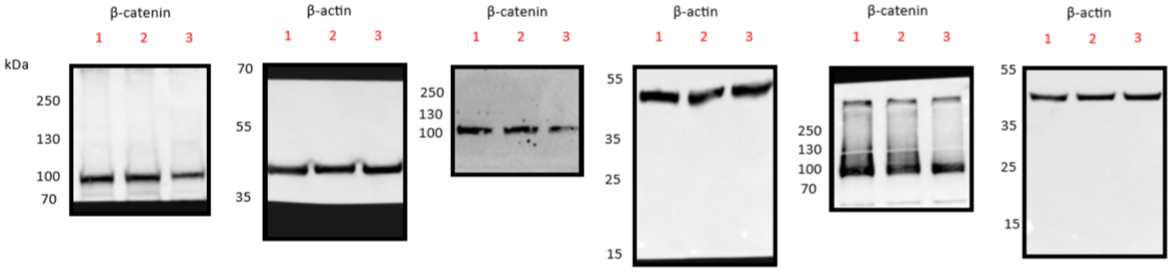

Ratio	FOLD1
1,129706	1
1,028483	0,910399
0,781897	0,692124

Ratio	FOLD2
0,890255	1
0,893151	1,003252
0,571315	0,641743

Ratio	FOLD3
1,726375	1
1,362596	0,789282
1,284431	0,744005

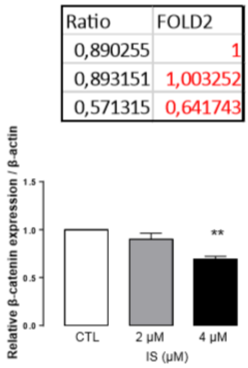

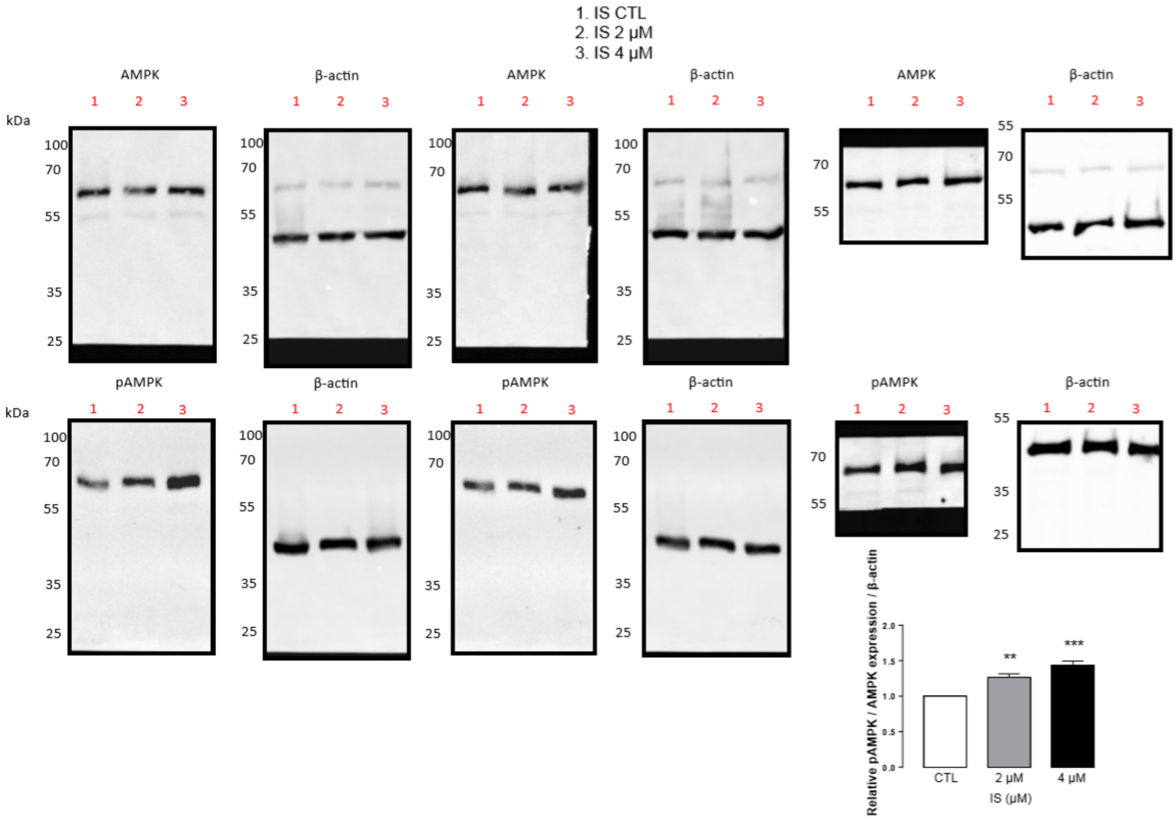

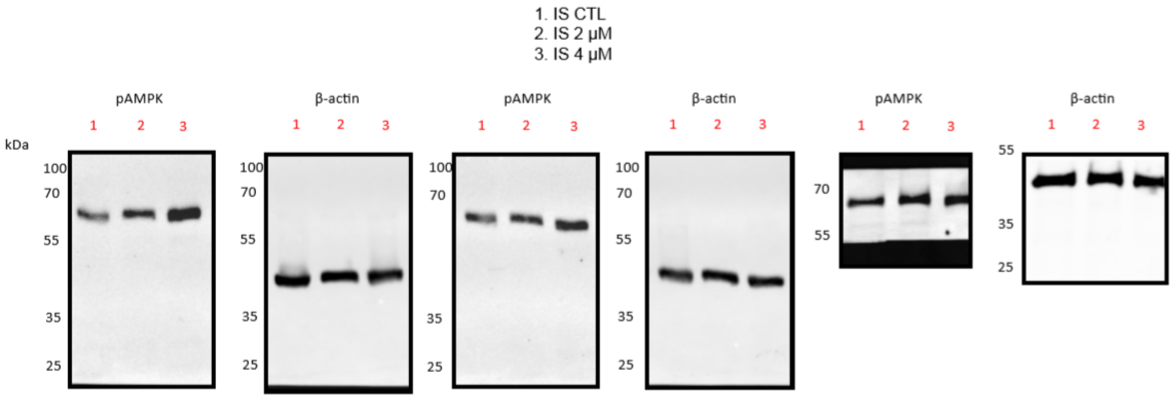

Ratio	FOLD1
0,846173	1
0,99673	1,177927
1,300797	1,53727

Ratio	FOLD2
1,100533	1
1,16456	1,058178
1,442892	1,311084

Ratio	FOLD3
0,740178	1
0,9185	1,240917
0,982191	1,326965

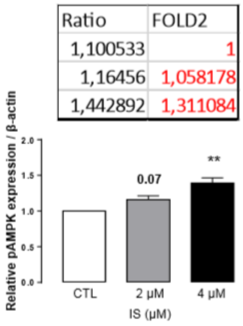

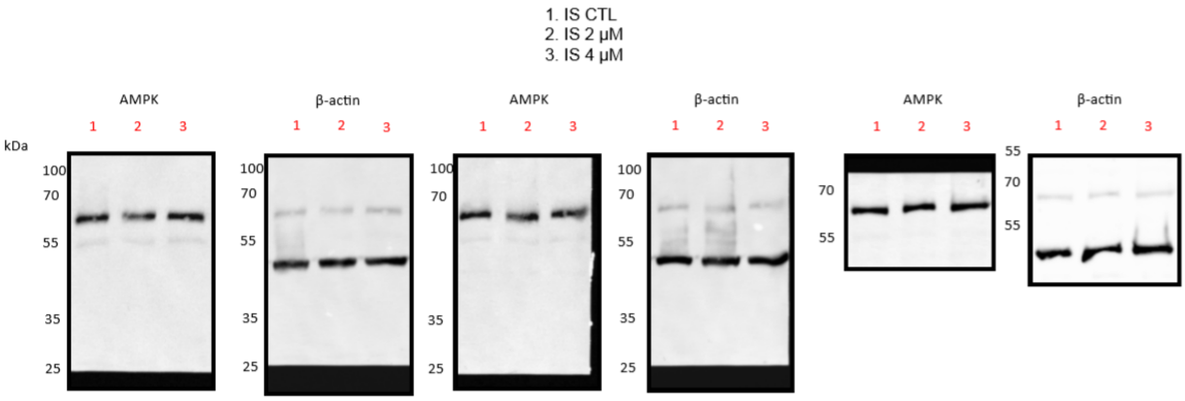

Ratio	FOLD1
1,055638	1
0,83676	0,792658
1,046525	0,991368

Ratio	FOLD2
0,830212	1
0,834804	1,005531
0,850008	1,023844

Ratio	FOLD3
1,269964	1
1,217653	0,958808
1,131911	0,891294

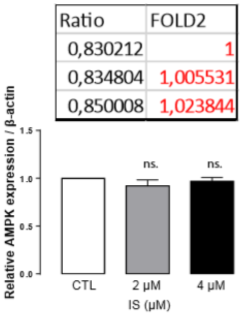

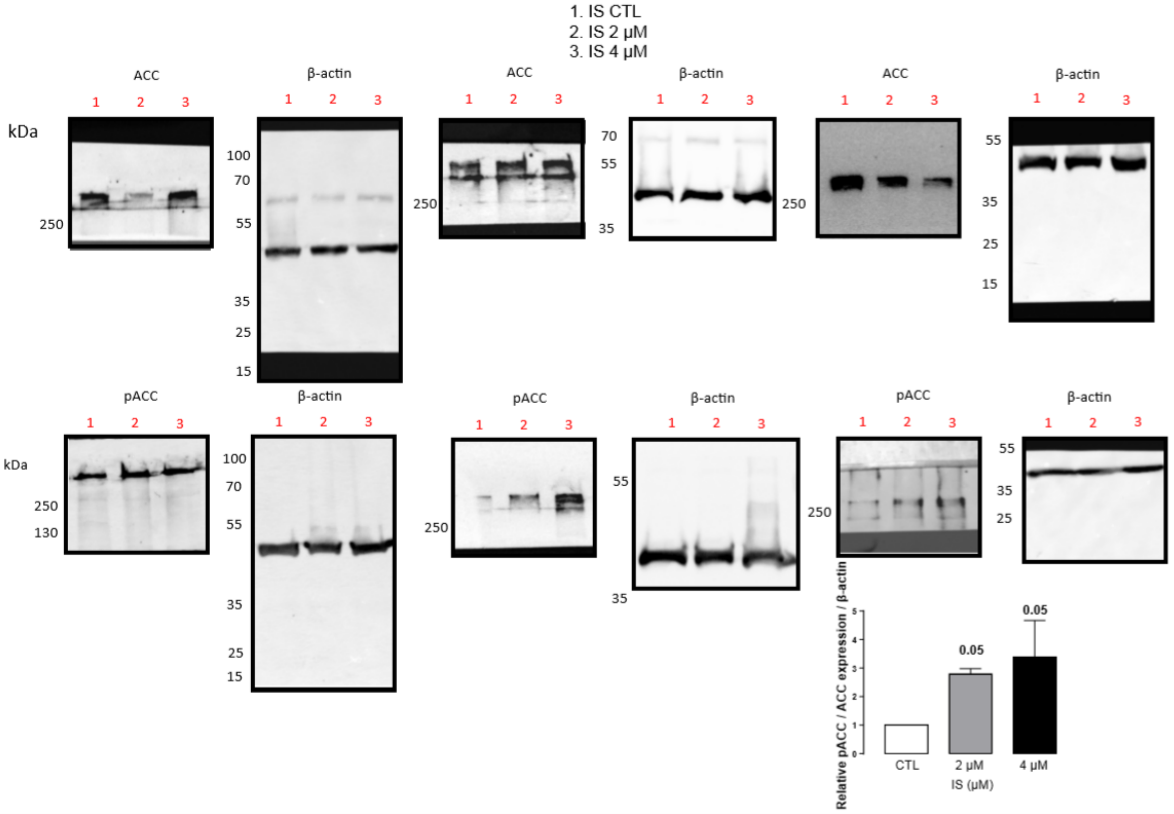

1. IS CTL
2. IS 2 µM
3. IS 4 µM

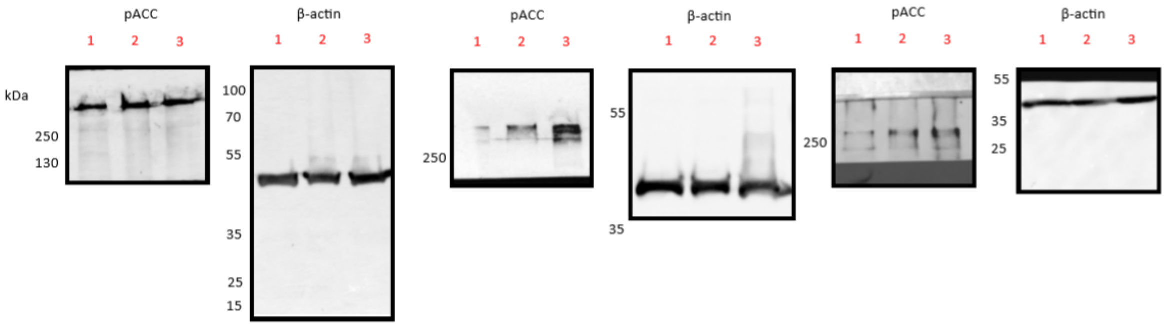

Ratio	FOLD1
1,156659	1
1,566244	1,354111
1,452683	1,25593

Ratio	FOLD2
0,212552	1
0,508412	2,391948
1,079342	5,078024

Ratio	FOLD3
0,31704	1
0,647604	2,042655
0,68453	2,159125

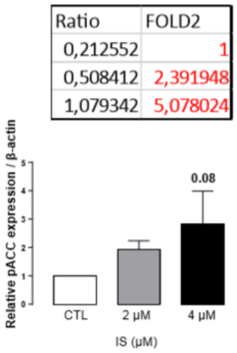

1. IS CTL
2. IS 2 µM
3. IS 4 µM

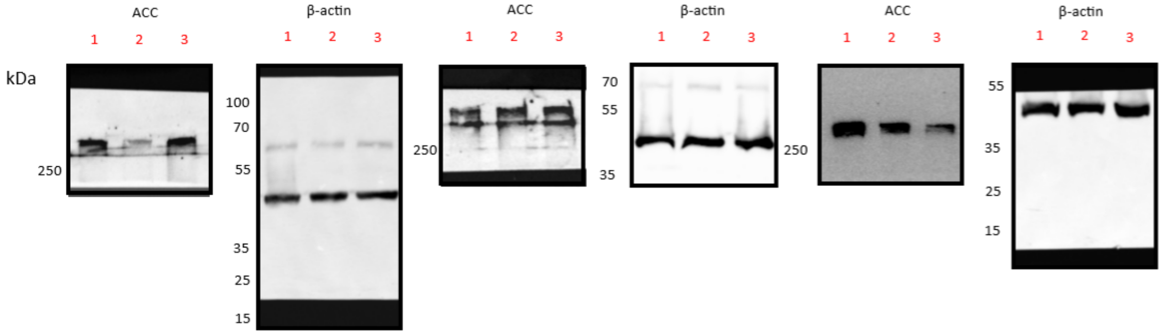

Ratio	FOLD1
1,010135	1
0,440473	0,436054
1,020582	1,010342

Ratio	FOLD2
1,324217	1
1,12833	0,852073
1,185876	0,89553

Ratio	FOLD3
1,039074	1
0,857648	0,825396
0,689568	0,663637

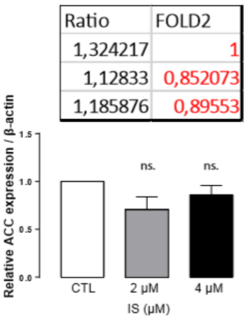

1. IS CTL
2. IS 2 µM
3. IS 4 µM

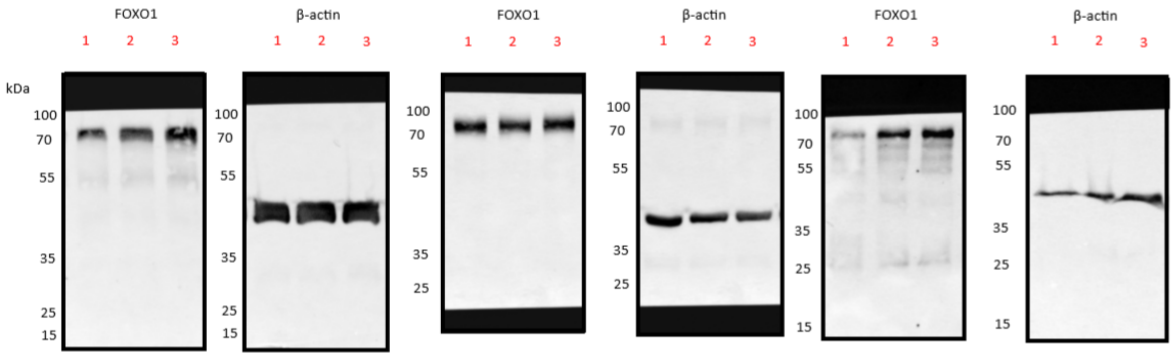

Ratio	FOLD1
0,636327	1
0,78858	1,239268
0,938178	1,474364

Ratio	FOLD2
1,237663	1
1,36989	1,106836
1,743809	1,408953

Ratio	FOLD3
0,830181	1
1,549707	1,86671
1,221166	1,470964

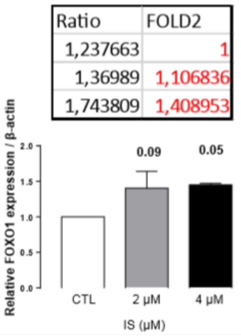

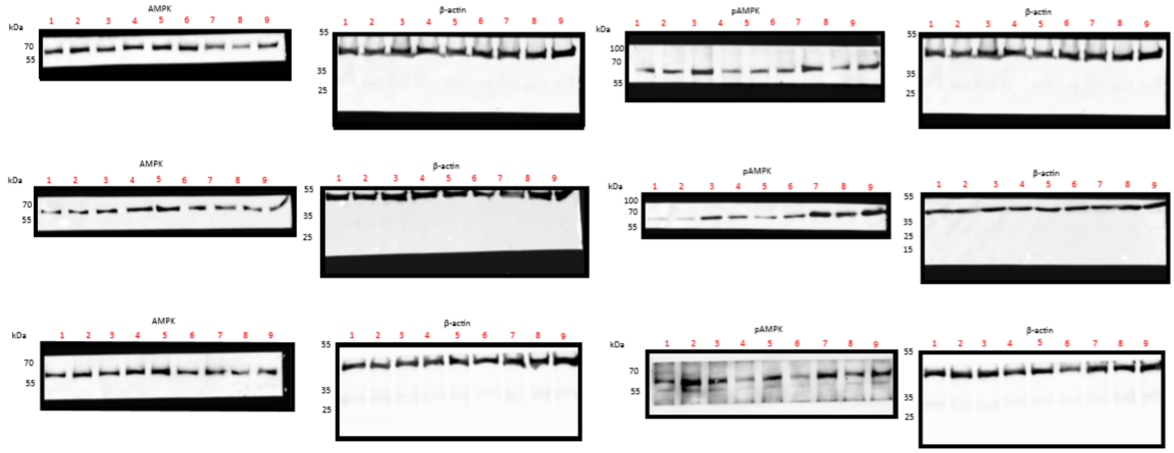

1. IS CTL
2. IS 2 µM
3. IS 4 µM
4. IS CTL
5. IS 2µM
6. IS 4 µM
7. IS CTL
8. IS 2 µM
9. IS 4 µM

CH223191 (AHRi)

Ketoconazole (PXRi)

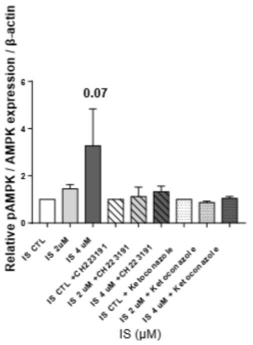

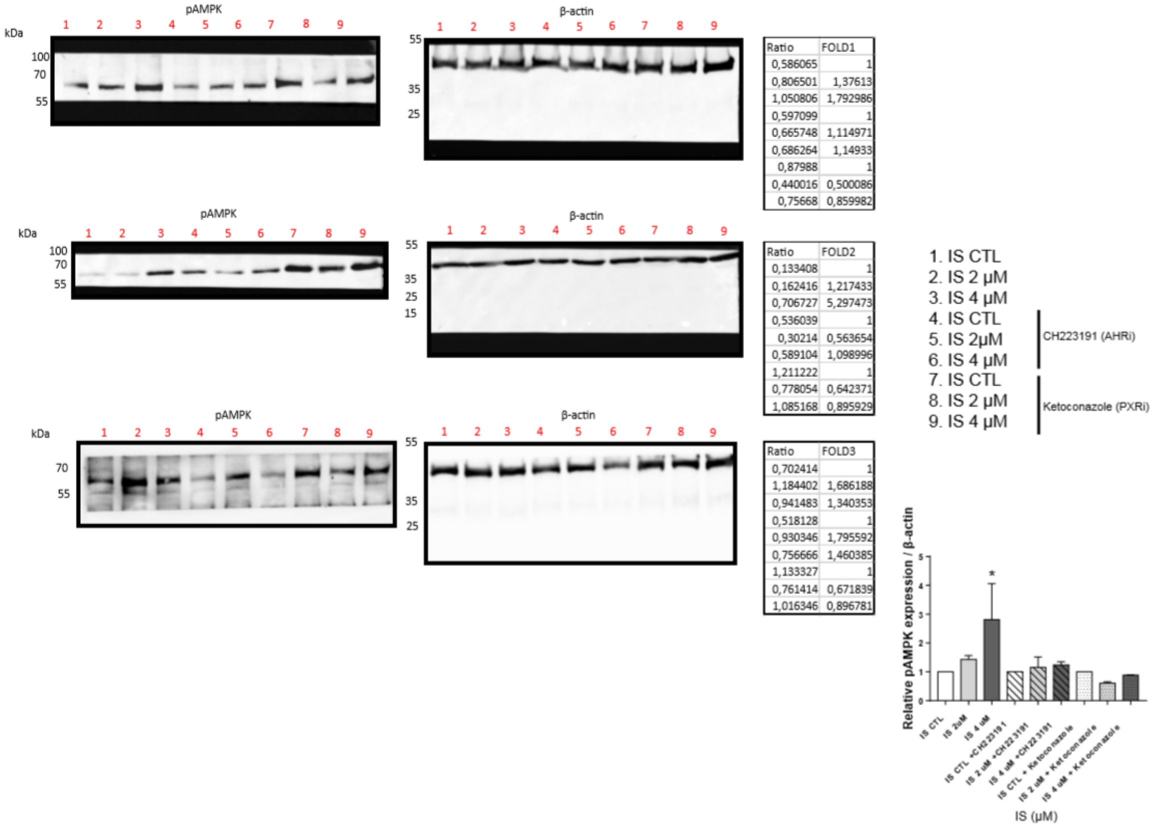

Ratio	FOLD1
0,586065	1
0,806501	1,37613
1,050806	1,792986
0,597099	1
0,665748	1,114971
0,686264	1,14933
0,87988	1
0,440016	0,500086
0,75668	0,859982

Ratio	FOLD2
0,133408	1
0,162416	1,217433
0,706727	5,297473
0,536039	1
0,30214	0,563654
0,589104	1,098996
1,211222	1
0,778054	0,642371
1,085168	0,895929

Ratio	FOLD3
0,702414	1
1,184402	1,686188
0,941483	1,340353
0,518128	1
0,930346	1,795592
0,756666	1,460385
1,133327	1
0,761414	0,671839
1,016346	0,896781

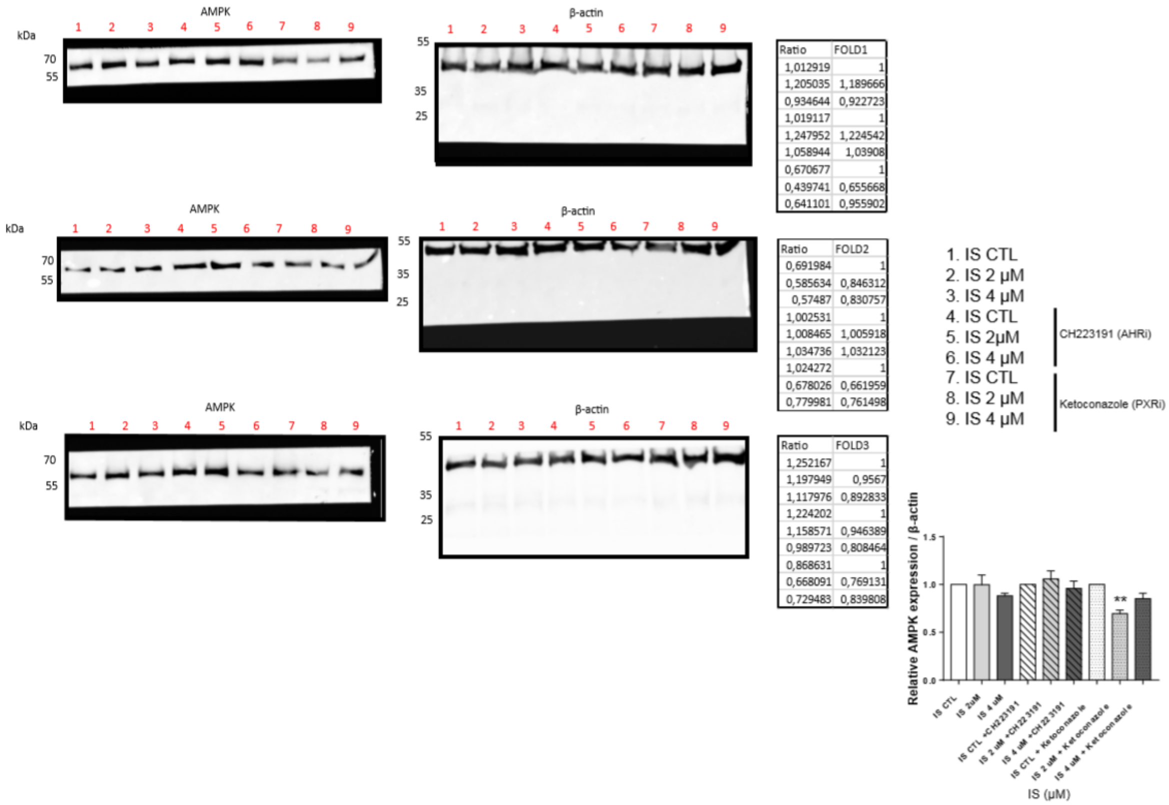

Ratio	FOLD1
1,012919	1
1,205035	1,189666
0,934644	0,922723
1,019117	1
1,247952	1,224542
1,058944	1,03908
0,670677	1
0,439741	0,655668
0,641101	0,955902

Ratio	FOLD2
0,691984	1
0,585634	0,846312
0,57487	0,830757
1,002531	1
1,008465	1,005918
1,034736	1,032123
1,024272	1
0,678026	0,661959
0,779981	0,761498

Ratio	FOLD3
1,252167	1
1,197949	0,9567
1,117976	0,892833
1,224202	1
1,158571	0,946389
0,989723	0,808464
0,868631	1
0,668091	0,769131
0,729483	0,839808

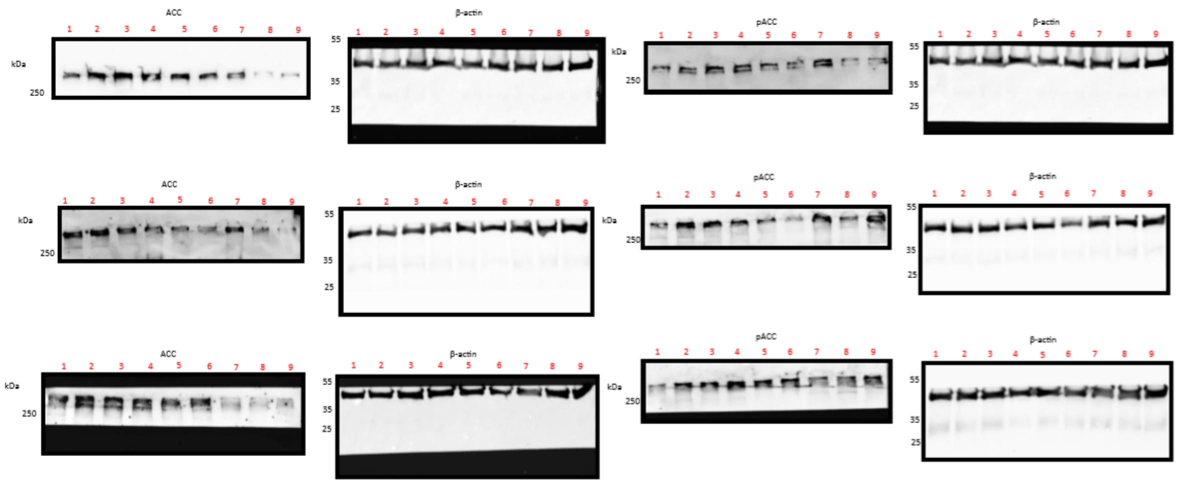

1. IS CTL
2. IS 2 μM
3. IS 4 μM
4. IS CTL
5. IS 2μM
6. IS 4 μM
7. IS CTL
8. IS 2 μM
9. IS 4 μM

CH223191 (AHRi) (4–6)

Ketoconazole (PXRi) (7–9)

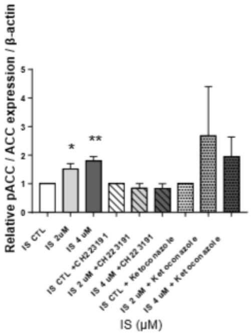

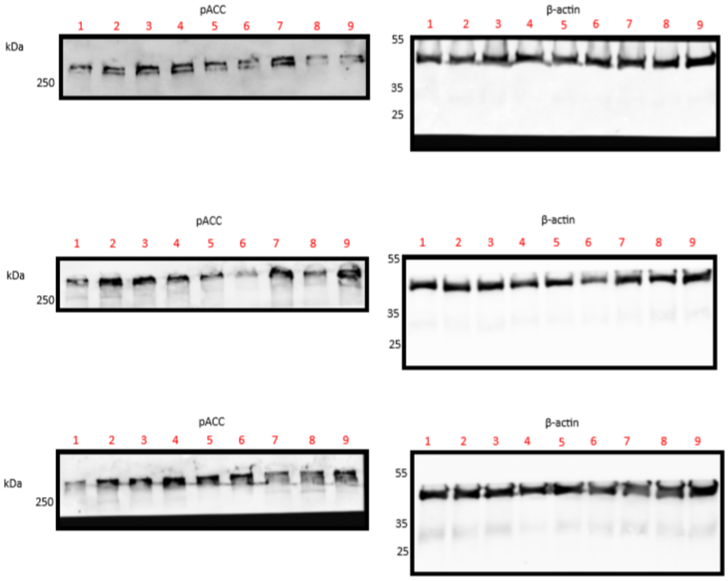

Ratio	FOLD1
0,835646	1
0,95484	1,142637
1,137491	1,361212
0,926828	1
0,972478	1,049255
0,655647	0,70741
0,953468	1
0,532709	0,558707
0,558458	0,585712

Ratio	FOLD2
0,569724	1
1,139237	1,99963
1,097562	1,926481
1,024364	1
0,689088	0,672698
0,58739	0,573419
1,030771	1
0,645382	0,626116
1,003976	0,974005

Ratio	FOLD3
0,44916	1
0,939248	2,091122
0,891	1,983706
1,122532	1
0,958073	0,853492
1,110989	0,989717
0,80566	1
0,813771	1,010067
0,804149	0,998125

1. IS CTL
2. IS 2 μM
3. IS 4 μM
4. IS CTL
5. IS 2μM
6. IS 4 μM
7. IS CTL
8. IS 2 μM
9. IS 4 μM

CH223191 (AHRi) (4–6)

Ketoconazole (PXRi) (7–9)

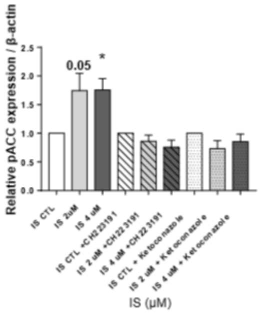

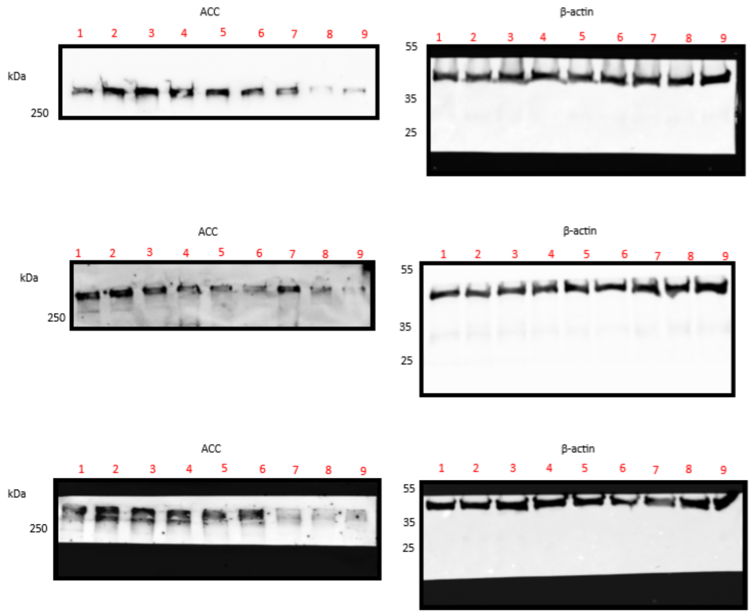

Ratio	FOLD1
1,019173	1
1,235446	1,212205
1,134918	1,113568
0,851801	1
1,177684	1,382582
0,812844	0,954265
0,59963	1
0,099087	0,165247
0,235893	0,393398

Ratio	FOLD2
1,131836	1
1,341833	1,185537
1,051554	0,92907
0,930772	1
0,779709	0,837701
0,554969	0,596246
0,881549	1
0,580318	0,658294
0,313305	0,355403

Ratio	FOLD3
0,88732	1
0,88519	0,9976
0,781917	0,881212
0,836323	1
0,771288	0,922236
1,165506	1,393607
0,439949	1
0,2513	0,571203
0,446038	1,01384

1. IS CTL
2. IS 2 μM
3. IS 4 μM
4. IS CTL
5. IS 2μM
6. IS 4 μM
7. IS CTL
8. IS 2 μM
9. IS 4 μM

(4–6) CH223191 (AHRi)
(7–9) Ketoconazole (PXRi)

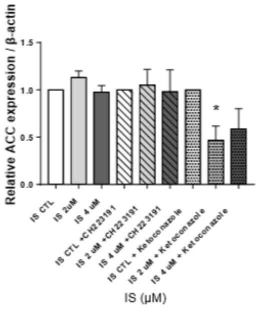

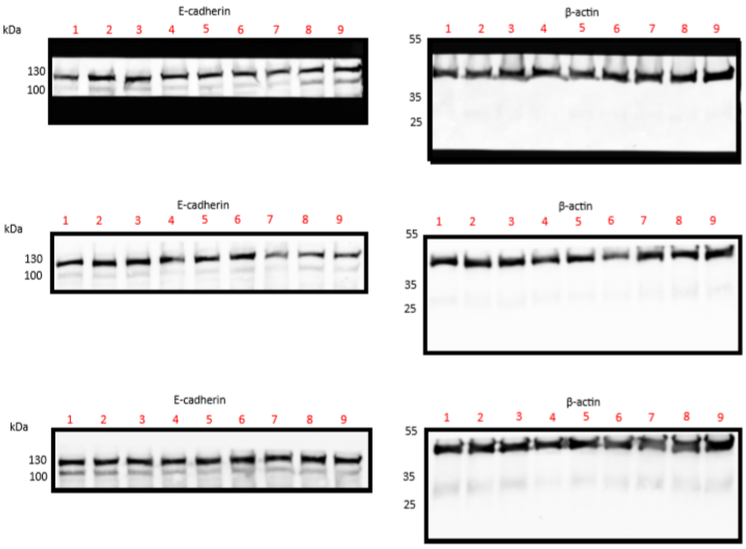

Ratio	FOLD1
0,835702	1
1,334903	1,597342
1,316719	1,575584
1,022428	1
1,117102	1,092598
0,932776	0,912314
0,77108	1
0,885529	1,148428
0,836988	1,085475

Ratio	FOLD2
0,74069	1
1,074946	1,451276
1,205893	1,628067
0,980846	1
0,909234	0,92699
1,524719	1,554494
0,569223	1
0,582009	1,022462
0,619371	1,0881

Ratio	FOLD3
0,866347	1
1,086052	1,253599
1,075969	1,24196
1,027956	1
0,954151	0,928202
1,230947	1,197471
1,321434	1
1,13866	0,861685
0,885106	0,669807

1. IS CTL
2. IS 2 μM
3. IS 4 μM
4. IS CTL
5. IS 2μM
6. IS 4 μM
7. IS CTL
8. IS 2 μM
9. IS 4 μM

(4–6) CH223191 (AHRi)
(7–9) Ketoconazole (PXRi)

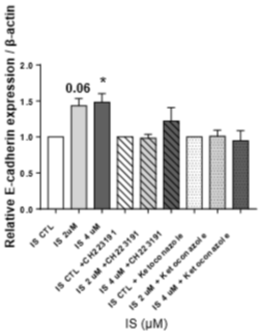

Figure S1. The whole western blot images of Figure 1B, Figure 3C, Figure 3D, and Figure 5B.
